# Effects of Nutritional Interventions During Pregnancy on Maternal and Neonatal Outcomes: A Systematic Review and Meta-Analysis of Randomized Controlled Trials

**DOI:** 10.3390/jcm15187297

**Published:** 2026-09-19

**Authors:** Zlatko Kirovakov, Andriana Jovanoska-Kirovakova, Pavel Dobrev, Nikolay Kostadinov, Plamen Penchev

**Affiliations:** 1 Faculty for Public Health and Health Care, Burgas State University “Prof. Dr. Assen Zlatarov”, 8010 Burgas, Bulgaria; kirovakov@yahoo.com (Z.K.); dr_dobrev@abv.bg (P.D.); n.kostadinov_m.d@abv.bg (N.K.); 2 Faculty of Medicine, Medical University of Plovdiv, 4002 Plovdiv, Bulgaria

**Keywords:** pregnancy, maternal nutrition, nutritional supplementation, birth weight, neonatal outcomes, meta-analysis

## Abstract

**Background:** Maternal undernutrition is associated with impaired fetal growth and adverse pregnancy outcomes, but the effects of food-based nutritional interventions during pregnancy remain uncertain. This systematic review and meta-analysis evaluated their effects on maternal and neonatal outcomes using evidence exclusively from randomized controlled trials (RCTs). **Methods:** PubMed, Embase, Scopus, and the Cochrane Library were searched from inception to 15 July 2026 for RCTs comparing food-based nutritional interventions with standard care, no supplementation, micronutrient supplementation, or an alternative nutritional intervention. Risk ratios (RRs) for binary outcomes were pooled using the Mantel–Haenszel method, while mean differences (MDs) for continuous outcomes were pooled using the inverse-variance method. Restricted maximum-likelihood random-effects models were applied, and heterogeneity was assessed using I^2^ and Cochran’s Q test. The protocol was registered in PROSPERO (CRD420261465279). **Results:** Six RCTs comprising 6245 participants were included, of whom 3150 (50%) received a nutritional intervention. Nutritional interventions increased birth weight (MD 51.49 g; 95% CI 22.87–80.11; I^2^ = 42.7%; *p* < 0.01) and birth length (MD 0.29 cm; 95% CI 0.14–0.45; I^2^ = 21.6%; *p* < 0.01). No significant effects were observed on birth head circumference (MD −0.14 cm; 95% CI −0.62 to 0.34; I^2^ = 73.9%; *p* = 0.57), low birth weight (RR 0.76; 95% CI 0.35–1.68; I^2^ = 64.2%; *p* = 0.28), stillbirth (RR 0.96; 95% CI 0.78–1.19; I^2^ = 0%; *p* = 0.61), or miscarriage (RR 0.83; 95% CI 0.35–1.97; I^2^ = 0%; *p* = 0.45). **Conclusions:** Food-based nutritional intervention strategies were associated with small statistically significant increases in birth weight and birth length; however, the clinical importance of differences of approximately 51 g and 0.29 cm remains uncertain. Evidence was inconclusive for low birth weight, head circumference, stillbirth, and miscarriage.

## 1. Introduction

Adequate maternal nutrition is essential for maintaining maternal health and supporting placental function, fetal growth, and development. Pregnancy increases requirements for energy, protein, vitamins, and minerals; when these requirements are not met, maternal undernutrition may contribute to inadequate gestational weight gain and adverse pregnancy outcomes, including fetal growth restriction, low birth weight, preterm birth, and perinatal mortality [1]. These risks are particularly important in low- and middle-income countries, where food insecurity and deficiencies of multiple nutrients frequently coexist.

Nutritional supplementation during pregnancy has therefore been proposed as a practical strategy for improving maternal and neonatal outcomes. Balanced protein–energy supplementation, generally defined as supplementation in which protein provides less than 25% of the total energy content, is intended to increase energy and protein intake without an excessive protein load. The World Health Organization recommends balanced energy and protein supplementation for pregnant women in undernourished populations to reduce the risks of stillbirth and small-for-gestational-age birth [2]. Previous systematic reviews suggest that these interventions may increase birth weight and reduce the occurrence of low birth weight, small-for-gestational-age birth, and stillbirth [3,4].

Nevertheless, the evidence remains inconclusive for several clinically relevant outcomes. Earlier reviews combined studies conducted across markedly different populations, nutritional settings, supplement formulations, intervention timings, and comparator types. The certainty of evidence has also been limited by methodological concerns and inconsistency between trials. Some reviews concentrated primarily on birth weight or infant anthropometry [4,5], whereas a recent synthesis included observational studies alongside randomized trials and focused predominantly on birth outcomes [6]. Consequently, the effects of prenatal nutritional supplementation across a broader range of maternal, fetal, and neonatal outcomes require further evaluation using evidence restricted to randomized controlled trials.

A systematic review and meta-analysis published in March 2026 evaluated the same clinical question and included six RCTs examining nutritional interventions during pregnancy [7]. That review reported statistically significant improvements in birth weight and birth length, as well as a lower risk of low birth weight and a lower mean neonatal head circumference. Our updated search did not identify additional eligible RCTs beyond the six trials included in that review. Therefore, the rationale for the present study is not an expansion of the evidence base through newly published trials, but an independent re-extraction and methodological re-evaluation of the available randomized evidence. This re-analysis was undertaken to verify the extracted outcome data, assess the influence of model selection, and provide a more detailed evaluation of heterogeneity, robustness, and potential effect modification.

This systematic review and meta-analysis therefore aimed to independently re-extract and re-analyse the available randomized evidence concerning food-based nutritional interventions during pregnancy. In addition to estimating pooled effects, we sought to evaluate the robustness and generalizability of the findings through consistent random-effects modelling, prediction intervals, RoB 2 assessment, prespecified subgroup analyses, risk-of-bias-stratified analyses, leave-one-out sensitivity analyses, and influence diagnostics.

## 2. Methods

### 2.1. Protocol Registration and Reporting Standards

This systematic review and meta-analysis followed the Cochrane Handbook and the PRISMA reporting guidelines [8,9]. Institutional review board approval was not required because only previously published data were used. The protocol was registered in PROSPERO (CRD420261465279). The full PRISMA checklist can be found in the Supplementary Materials (Figure S32).

### 2.2. Eligibility Criteria

Studies were eligible if they: (1) enrolled pregnant women, irrespective of age, parity, baseline nutritional status, geographical setting, or gestational age at enrolment; studies initiating supplementation before conception were eligible provided that supplementation continued during pregnancy; (2) evaluated a food-based nutritional intervention intended to improve maternal nutritional status, including balanced protein–energy supplements, energy-dense biscuits, lipid-based nutrient supplements, locally produced supplementary foods, or nutrient-rich food-based supplements, administered alone or alongside routine antenatal supplementation; (3) included an eligible comparator, defined as placebo or no nutritional supplementation, standard or usual antenatal care, iron–folic acid or other micronutrient supplementation, or an alternative or lower-intensity nutritional intervention; (4) reported at least one outcome of interest, including birth weight, low birth weight, birth length, head circumference, miscarriage, and stillbirth; (5) used a randomized controlled design; and (6) administered the intervention during pregnancy and reported relevant outcomes during pregnancy, at delivery, or during the neonatal period. No minimum intervention or follow-up duration was imposed.

Studies were excluded if they: (1) were non-human studies or did not involve pregnant women; (2) evaluated supplementation administered exclusively during the postpartum period; (3) focused exclusively on women with HIV infection or another specific medical condition for which results applicable to the general pregnant population could not be extracted separately; (4) evaluated dietary counselling or education without nutritional supplementation, micronutrient-only supplementation as the experimental intervention, or interventions that did not contain an eligible food-based or protein–energy component; (5) had no eligible comparator group; (6) did not report a maternal, pregnancy, fetal, or neonatal outcome of interest; (7) were observational or non-randomized studies, reviews, protocols, editorials, case reports, conference abstracts, dissertations, or letters without original randomized trial results; or (8) represented duplicate publications or overlapping trial populations. For duplicate or overlapping reports, the most complete report was retained, while relevant companion publications were linked to the same trial.

For this review, a food-based nutritional intervention was operationally defined as an orally consumed food, snack, biscuit, paste, or lipid-based formulation that provided energy and macronutrients, with or without micronutrient fortification. Accordingly, lipid-based nutrient supplements and ready-to-use supplementary foods were considered food-based because they were delivered within an energy-containing food matrix and provided protein and/or fat in addition to micronutrients. Interventions consisting exclusively of isolated micronutrients in tablets, capsules, or non-energy-containing formulations were excluded.

Trials evaluating multicomponent intervention packages were eligible when the experimental intervention contained an eligible food-based nutritional component. For such trials, the extracted effect was interpreted as the effect of the complete randomized intervention package rather than as the isolated effect of its nutritional component.

### 2.3. Search Strategy and Data Extraction

We systematically searched MEDLINE via PubMed, Embase, Scopus, and the Cochrane Central Register of Controlled Trials (CENTRAL) from database inception to 15 July 2026 with the following search strategy:

(pregnancy OR pregnant OR antenatal OR prenatal OR maternal) AND (“dietary supplement” OR “nutritional supplement” OR “maternal nutrition supplement” OR “balanced energy protein” OR “balanced protein energy” OR “protein energy supplement” OR “protein-energy supplement” OR “energy protein supplement” OR “energy-protein supplement” OR “food supplement” OR “supplementary food” OR “supplementary feeding” OR “lipid-based nutrient supplement” OR “lipid based nutrient supplement” OR LNS OR “energy-dense biscuit” OR “energy dense biscuit” OR “nutrient-rich food” OR “nutrient rich food” OR “food-based supplement” OR “food based supplement” OR “fortified food” OR “ready-to-use supplemental food”) AND (“randomized controlled trial” OR “controlled clinical trial” OR random OR trial OR placebo OR cluster-random).

Searches were restricted to English-language publications, and grey literature was excluded. These restrictions were pragmatic: resources for reliable translation and appraisal of non-English full texts were unavailable, while grey-literature reports could not consistently provide the methodological and outcome information required for eligibility verification, data extraction, and RoB 2 assessment. After duplicate removal, two reviewers (Z.K and A.J.K) independently screened titles and abstracts and subsequently assessed potentially eligible full-text articles using predefined eligibility criteria in Rayyan [10]. Data were independently extracted using a standardized data-extraction form. Disagreements at any stage were resolved through discussion and consensus or, when necessary, consultation with a third reviewer (P.P). The reference lists of included studies and relevant systematic reviews were manually examined, and forward citation tracking was undertaken to identify additional eligible records.

### 2.4. Endpoints and Subgroup Analyses

The primary outcomes were birth weight, birth length, birth head circumference, low birth weight, stillbirth, and miscarriage. Prespecified subgroup analyses according to maternal undernutrition eligibility (targeted versus non-targeted populations) and comparator type (active versus inactive comparator) were conducted to explore clinical heterogeneity and potential effect modification. An additional stratified analysis according to overall risk of bias (low versus non-low risk) was performed as a methodological sensitivity analysis rather than as a clinical subgroup analysis.

Low birth weight was defined as birth weight < 2500 g. Miscarriage and stillbirth were extracted according to the definitions provided by the original investigators. Where explicit definitions were available, miscarriage referred to pregnancy loss at or before 20 weeks of gestation, whereas stillbirth referred to delivery of an infant showing no signs of life at or after 20 weeks. However, gestational-age thresholds were not reported consistently across all included trials. Therefore, definitions of miscarriage and stillbirth were not fully uniform or verifiable, and the corresponding pooled estimates were interpreted cautiously.

### 2.5. Quality Assessment

The risk of bias was assessed using the Cochrane Collaboration’s tool for assessing the risk of bias in randomized studies of interventions (RoB2) [11]. The RoB2 tool categorizes the risk of bias as low, some concerns, or high. Two authors (P.D. and N.K.) independently performed the assessments, resolving disagreements through consensus. Small-study effects were explored descriptively using contour-enhanced funnel plots and the trim-and-fill method. Because fewer than 10 studies contributed to every outcome, Egger’s test was not performed, and the funnel plots and trim-and-fill estimates were regarded as exploratory rather than as reliable assessments of publication bias [8].

### 2.6. Statistical Analysis

Risk ratios (RRs) with 95% confidence intervals (CIs) were pooled for binary outcomes using the Mantel–Haenszel method [12]. Mean differences (MDs) with 95% CIs were pooled for continuous outcomes using the inverse-variance method [13]. Random-effects models were applied to all outcomes, with between-study variance (τ^2^) estimated using restricted maximum likelihood (REML) [14,15]. Heterogeneity was evaluated using Cochran’s Q test and quantified using the I^2^ statistic. When estimable, 95% prediction intervals were calculated to describe the expected range of effects in a comparable future study. Two-sided *p*-values < 0.05 were considered statistically significant.

Prespecified subgroup analyses according to comparator type and maternal undernutrition eligibility were performed to explore heterogeneity and potential effect modification. Stratification by overall risk of bias was undertaken as a methodological sensitivity analysis. Leave-one-out (LOO) sensitivity analyses were conducted to assess the robustness of pooled estimates. Baujat plots were generated to identify influential studies by displaying each study’s contribution to overall heterogeneity on the x-axis and its influence on the pooled effect on the y-axis. Analyses were conducted using R version 4.3.1 with the “meta” and “metafor” packages [16].

## 3. Results

### 3.1. Study Selection and Baseline Characteristics

**Figure 1:**
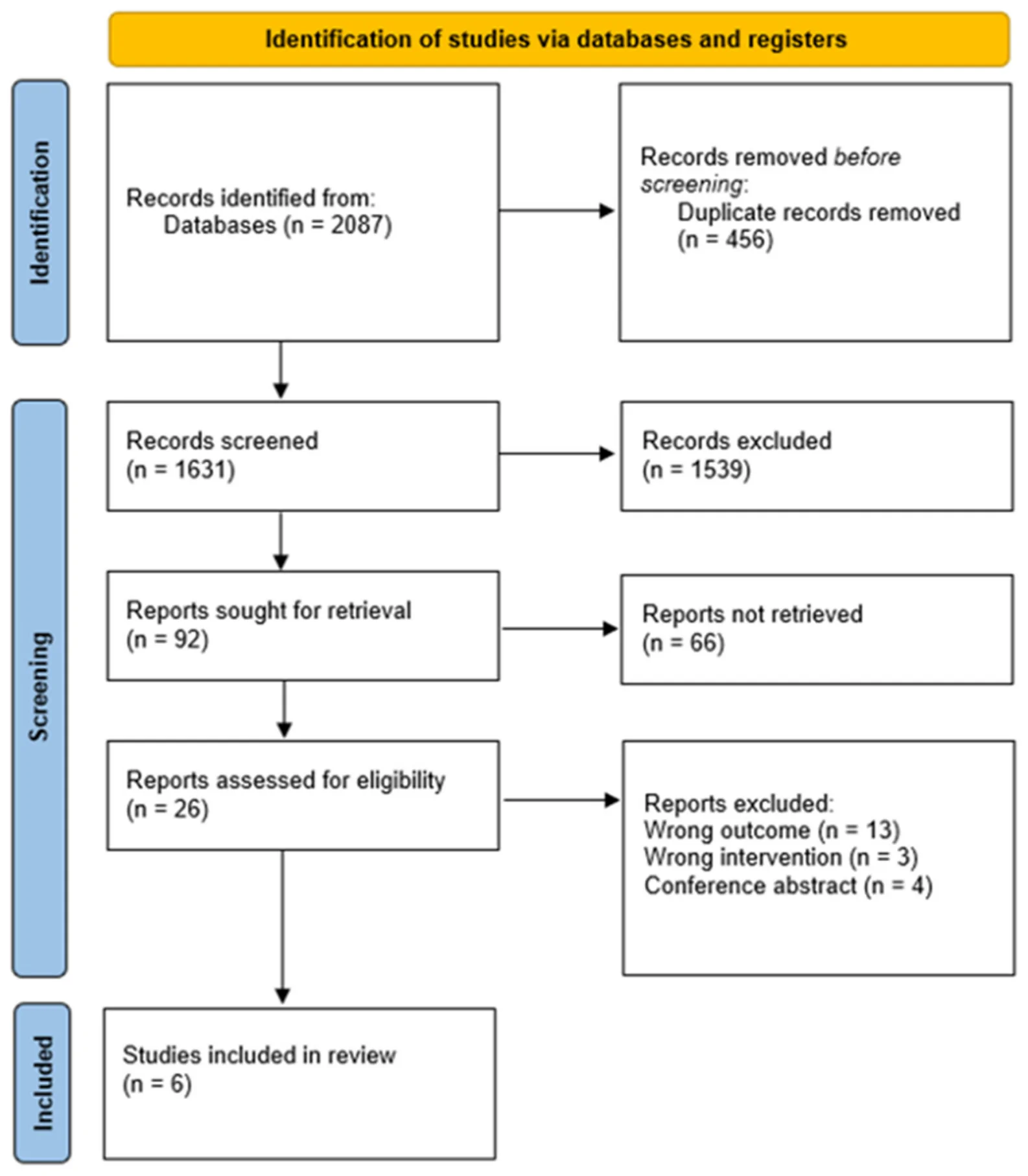
PRISMA flow diagram and study selection process.

The database searches identified 2087 records. After the removal of 456 duplicates, 1631 titles and abstracts were screened, of which 1539 were excluded. Ninety-two reports were sought for retrieval; 66 could not be retrieved, leaving 26 full-text reports that were assessed for eligibility. Twenty reports were excluded because of an ineligible outcome (n = 13), an ineligible intervention (n = 3), or publication as a conference abstract (n = 4). Six RCTs met the eligibility criteria and were included in the systematic review and meta-analysis (Figure 1) [17,18,19,20,21,22].

The six included RCTs comprised 6245 participants, of whom 3150 (50.4%) received a nutritional intervention and 3095 (49.6%) received a control intervention. Because outcome availability and the number of participants assessed varied across trials, the outcome-specific pooled sample ranged from 1711 participants for miscarriage to 4111 participants for stillbirth. Five trials reported birth weight, four reported birth length, head circumference, and stillbirth, and three reported low birth weight and miscarriage. The principal characteristics of the included studies, interventions, comparators, and study populations are summarized in Table 1.

### 3.2. Meta-Analysis Results

#### 3.2.1. Birth Weight

**Figure 2:**
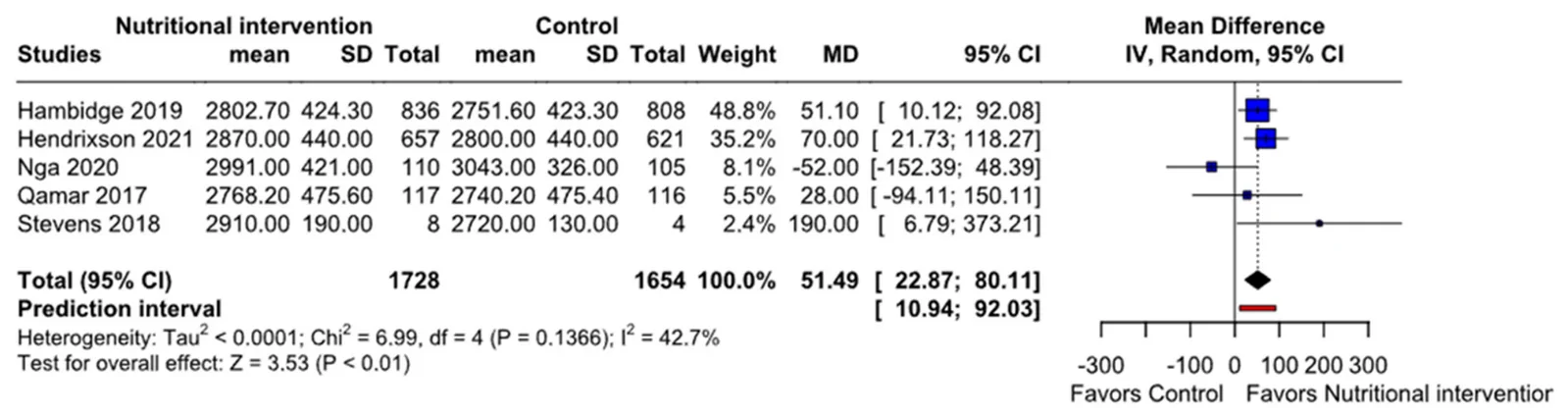
Forest plot comparing nutritional interventions with controls, showing a statistically significant increase in birth weight with nutritional interventions [18,19,20,21,22].

Five RCTs involving 3382 participants showed that nutritional interventions were associated with higher birth weight compared with controls (MD 51.49 g, 95% CI 22.87–80.11; *p* < 0.01), with moderate between-study heterogeneity (I^2^ = 42.7%) (Figure 2).

LOO analysis produced consistently positive effect estimates (MD range 36.18–60.64 g). The omission of Nga 2020 [21] eliminated heterogeneity (I^2^ = 0%) (Figure S1). The Baujat plot identified Nga 2020 [21] as the largest contributor to heterogeneity and the most influential study, while Hendrixson 2021 [22] also had substantial influence on the pooled estimate (Figure S2).

#### 3.2.2. Birth Length

**Figure 3:**
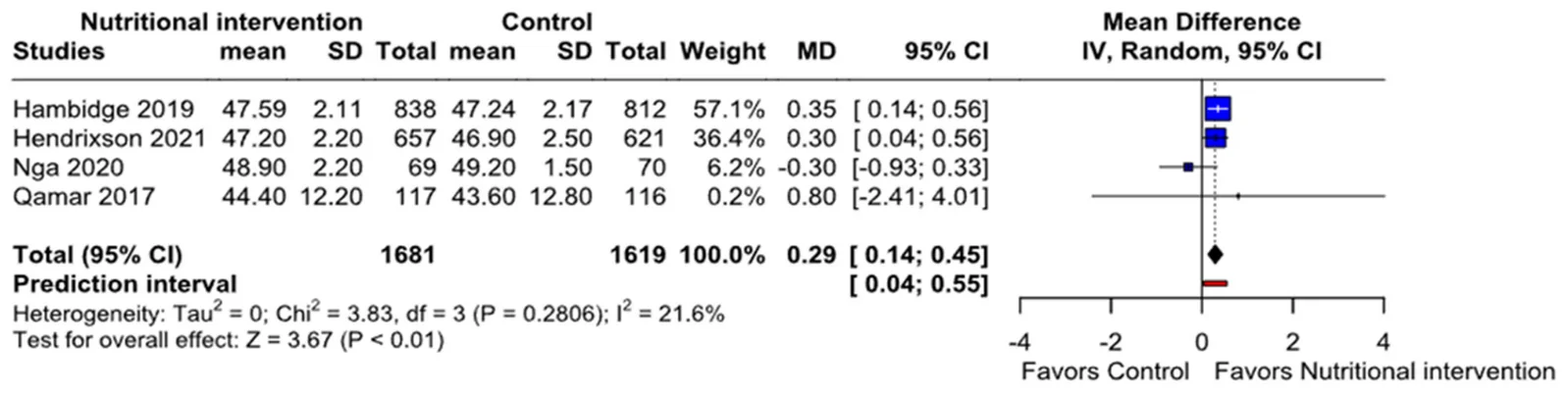
Forest plot comparing nutritional interventions with controls, showing a statistically significant increase in birth length with nutritional interventions [18,19,21,22].

Four RCTs involving 3300 participants showed that nutritional interventions were associated with greater birth length compared with controls (MD 0.29 cm, 95% CI 0.14–0.45; *p* < 0.01), with low heterogeneity (I^2^ = 21.6%) (Figure 3).

LOO estimates remained positive (MD range 0.10–0.33 cm), although statistical significance was lost after omitting Hambidge 2019 [19] or Hendrixson 2021 [22]. Omission of Nga 2020 [21] eliminated heterogeneity (I^2^ = 0%) (Figure S3). The Baujat plot identified Nga 2020 [21] as the largest contributor to heterogeneity, whereas Hambidge 2019 [19] exerted the greatest influence on the pooled estimate (Figure S4).

#### 3.2.3. Birth Head Circumference

**Figure 4:**
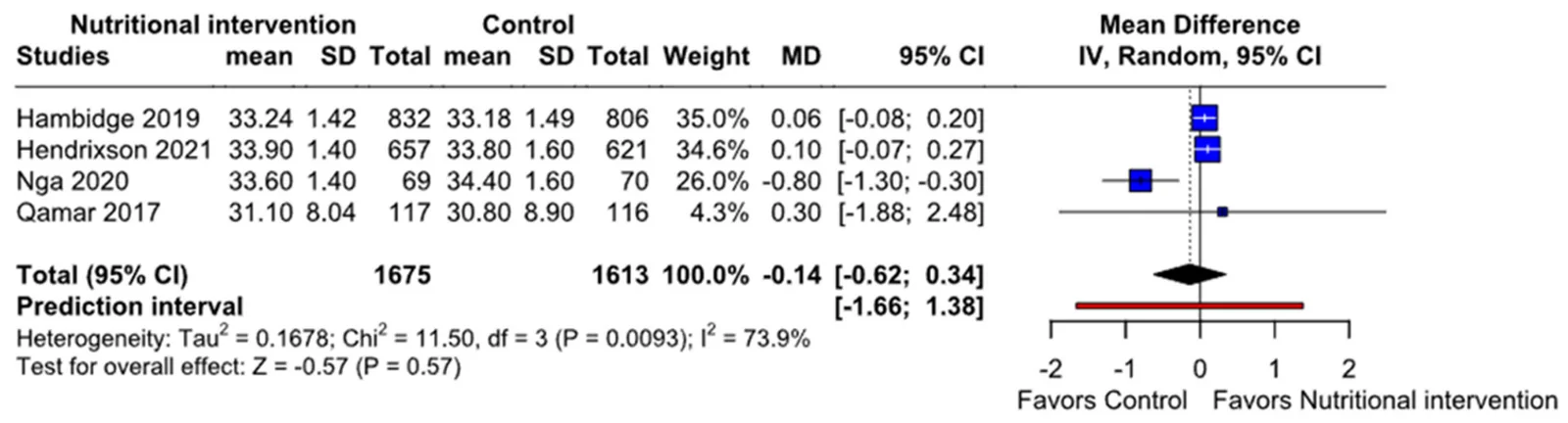
Forest plot comparing nutritional interventions with controls, showing no statistically significant difference in birth head circumference and substantial between-study heterogeneity [18,19,21,22].

Four RCTs involving 3288 participants showed no significant difference in birth head circumference between nutritional interventions and controls (MD −0.14 cm, 95% CI −0.62 to 0.34; *p* = 0.57), with substantial heterogeneity (I^2^ = 73.9%) (Figure 4).

LOO analysis showed no significant effect in any iteration (MD range −0.27 to 0.08 cm). Omission of Nga 2020 [21] eliminated heterogeneity (I^2^ = 0%), identifying it as the principal source of between-study variability (Figure S5). The Baujat plot identified Nga 2020 [21] as both the largest contributor to heterogeneity and the most influential study, while Hendrixson 2021 [22] had a smaller influence on the pooled estimate (Figure S6).

#### 3.2.4. Low Birth Weight

**Figure 5:**
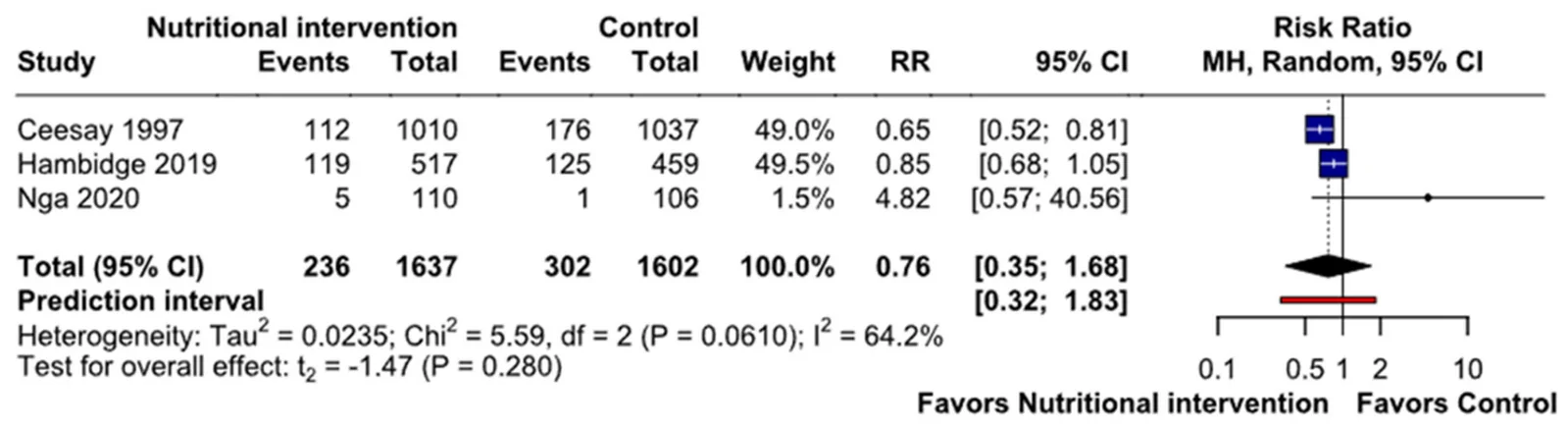
Forest plot comparing nutritional interventions with controls, showing no statistically significant difference in the risk of low birth weight and substantial between-study heterogeneity [17,19,21].

Three RCTs involving 3239 participants showed no significant difference in the risk of low birth weight between nutritional interventions and controls (RR 0.76, 95% CI 0.35–1.68; *p* = 0.28), with substantial heterogeneity (I^2^ = 64.2%) (Figure 5).

LOO analysis remained non-significant in every iteration (RR range 0.74–1.44), with substantial heterogeneity persisting (I^2^ range 60.6–70.1%) and marked imprecision after omitting Ceesay 1997 [17] or Hambidge 2019 [19] (Figure S7). The Baujat plot identified Nga 2020 [21] as the largest contributor to heterogeneity, whereas Ceesay 1997 [17] exerted the greatest influence on the pooled estimate (Figure S8).

#### 3.2.5. Stillbirth

**Figure 6:**
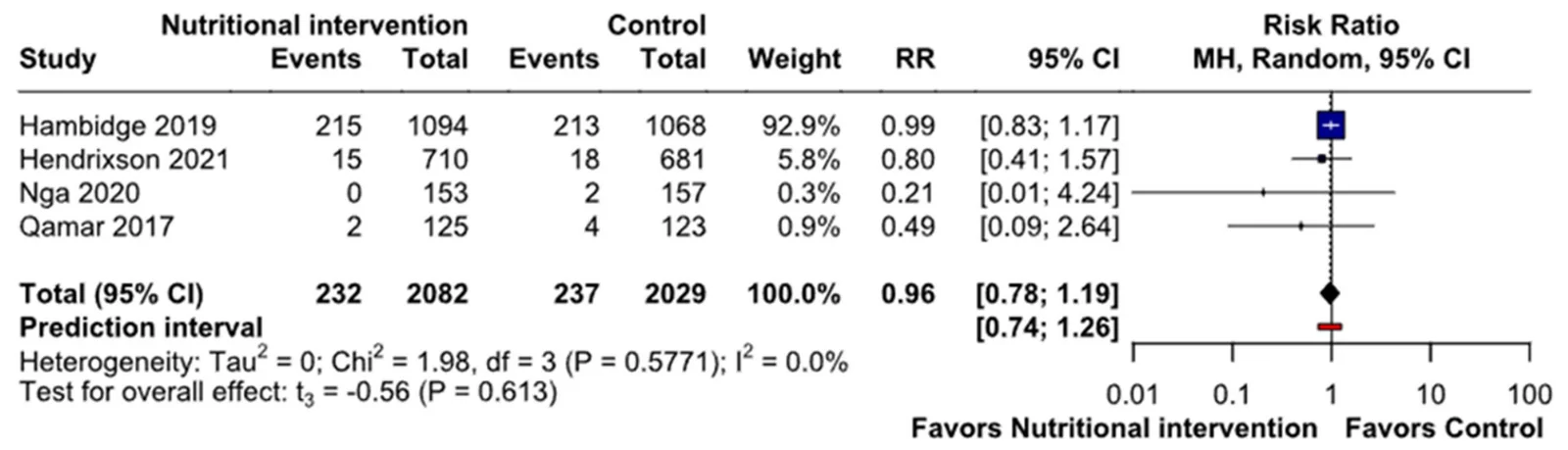
Forest plot comparing nutritional interventions with controls, showing no statistically significant difference in the risk of stillbirth and no observed between-study heterogeneity [18,19,21,22].

Four RCTs involving 4111 participants showed no significant difference in the risk of stillbirth between nutritional interventions and controls (RR 0.96, 95% CI 0.78–1.19; *p* = 0.61), with no observed heterogeneity (I^2^ = 0%) (Figure 6).

LOO analysis remained non-significant in every iteration (RR range 0.71–0.97), with no observed heterogeneity throughout (I^2^ = 0%) (Figure S9).

#### 3.2.6. Miscarriage

**Figure 7:**
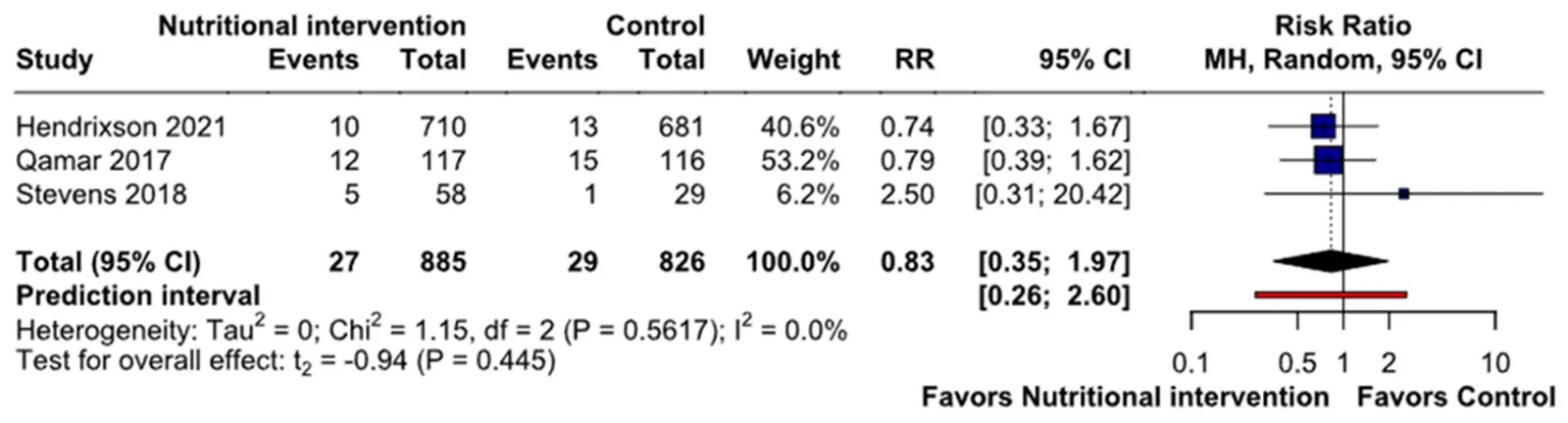
Forest plot comparing nutritional interventions with controls, showing no statistically significant difference in the risk of miscarriage and no observed between-study heterogeneity [18,20,22].

Three RCTs involving 1711 participants showed no significant difference in the risk of miscarriage between nutritional interventions and controls (RR 0.83, 95% CI 0.35–1.97; *p* = 0.45), with no observed heterogeneity (I^2^ = 0%) (Figure 7).

LOO analysis remained non-significant in every iteration (RR range 0.77–0.91), with negligible heterogeneity (I^2^ range 0–2.8%) and marked imprecision after omitting Hendrixson 2021 [22] or Qamar 2017 [18] (Figure S10).

### 3.3. Subgroup and Sensitivity Analyses

Subgroup findings were interpreted primarily according to the formal tests for interaction. Statistical significance within one subgroup but not another was not considered evidence of effect modification when the between-subgroup difference was not statistically significant.

#### 3.3.1. Maternal Undernutrition Eligibility

Birth weight. Among trials targeting undernourished women (three RCTs; n = 1523), nutritional interventions increased birth weight (MD 71.44 g, 95% CI 27.84–115.04; I^2^ = 4.7%). No significant effect was observed in non-targeted populations (two RCTs; n = 1859; MD 10.15 g, 95% CI −88.72 to 109.03; I^2^ = 71.2%). However, the between-subgroup difference was not statistically significant (*p* = 0.27) (Figure S11).

**Birth length.** Among trials targeting undernourished women (two RCTs; n = 1511), nutritional interventions increased birth length (MD 0.30 cm, 95% CI 0.05–0.56; I^2^ = 0%). No significant effect was observed in non-targeted populations (two RCTs; n = 1789; MD 0.10 cm, 95% CI −0.53 to 0.72; I^2^ = 73.2%). However, the between-subgroup difference was not statistically significant (*p* = 0.54) (Figure S12).

**Birth head circumference.** No significant effect on head circumference was observed in either targeted populations (two RCTs; n = 1511; MD 0.10 cm, 95% CI −0.06 to 0.27; I^2^ = 0%) or non-targeted populations (two RCTs; n = 1777; MD −0.34 cm, 95% CI −1.18 to 0.50; I^2^ = 90.5%). The between-subgroup difference was not statistically significant (*p* = 0.32) (Figure S13).

**Stillbirth.** No significant effect on stillbirth was observed in either targeted populations (two RCTs; n = 1639; RR 0.75, 95% CI 0.09–6.34; I^2^ = 0%) or non-targeted populations (two RCTs; n = 2472; RR 0.96, 95% CI 0.07–12.34; I^2^ = 2.7%). The between-subgroup difference was not statistically significant (*p* = 0.34) (Figure S14).

#### 3.3.2. Comparator Type

Birth weight. Nutritional interventions increased birth weight in trials with active comparators (two RCTs; n = 1511; MD 64.32 g, 95% CI 19.44–109.21; I^2^ = 0%), whereas no significant effect was observed with inactive comparators (three RCTs; n = 1871; MD 44.31 g, 95% CI −65.61 to 154.23; I^2^ = 67.0%). The between-subgroup difference was not statistically significant (*p* = 0.74) (Figure S15).

**Birth length.** Nutritional interventions increased birth length in trials with active comparators (two RCTs; n = 1511; MD 0.30 cm, 95% CI 0.05–0.56; I^2^ = 0%), whereas no significant effect was observed with inactive comparators (two RCTs; n = 1789; MD 0.10 cm, 95% CI −0.53 to 0.72; I^2^ = 73.2%). The between-subgroup difference was not statistically significant (*p* = 0.54) (Figure S16).

**Birth head circumference.** No significant effect on head circumference was observed in trials with either active comparators (two RCTs; n = 1511; MD 0.10 cm, 95% CI −0.06 to 0.27; I^2^ = 0%) or inactive comparators (two RCTs; n = 1777; MD −0.34 cm, 95% CI −1.18 to 0.50; I^2^ = 90.5%). The between-subgroup difference was not statistically significant (*p* = 0.32) (Figure S17).

**Stillbirth.** No significant effect on stillbirth was observed in trials with either active comparators (two RCTs; n = 1639; RR 0.75, 95% CI 0.09–6.34; I^2^ = 0%) or inactive comparators (two RCTs; n = 2472; RR 0.96, 95% CI 0.07–12.34; I^2^ = 2.7%). The between-subgroup difference was not statistically significant (*p* = 0.34) (Figure S18).

**Miscarriage.** No significant effect on miscarriage was observed in trials with active comparators (two RCTs; n = 1624; RR 0.77, 95% CI 0.49–1.21; I^2^ = 0%) or in the single trial with an inactive comparator (n = 87; RR 2.50, 95% CI 0.31–20.42). The between-subgroup difference was not statistically significant (*p* = 0.27) (Figure S19).

#### 3.3.3. Overall Risk of Bias

**Birth weight.** In sensitivity analyses stratified by overall risk of bias, studies at low risk showed increased birth weight (two RCTs; n = 2922; MD 59.02 g, 95% CI 27.78–90.25; I^2^ = 0%), whereas studies at non-low risk showed no significant effect (three RCTs; n = 460; MD 36.81 g, 95% CI −89.35 to 162.97; I^2^ = 61.9%). The between-subgroup difference was not statistically significant (*p* = 0.74) (Figure S20).

**Birth length.** Studies at low risk of bias showed increased birth length (two RCTs; n = 2928; MD 0.33 cm, 95% CI 0.17–0.49; I^2^ = 0%), whereas studies at non-low risk showed no significant effect (two RCTs; n = 372; MD −0.26 cm, 95% CI −0.87 to 0.36; I^2^ = 0%). The between-subgroup difference did not reach statistical significance (*p* = 0.07) (Figure S21).

**Birth head circumference.** Studies at low risk of bias showed no significant effect on head circumference (two RCTs; n = 2916; MD 0.08 cm, 95% CI −0.03 to 0.18; I^2^ = 0%), whereas studies at non-low risk showed a significant reduction (two RCTs; n = 372; MD −0.75 cm, 95% CI −1.23 to −0.26; I^2^ = 0%). The between-subgroup difference was statistically significant (*p* = 0.001) (Figure S22).

**Low birth weight.** No significant effect on low birth weight was observed among studies at low risk of bias (two RCTs; n = 3023; RR 0.74, 95% CI 0.14–3.82; I^2^ = 62.4%) or in the single study at non-low risk (n = 216; RR 4.82, 95% CI 0.57–40.56). The between-subgroup difference was not statistically significant (*p* = 0.09) (Figure S23).

**Stillbirth.** No significant effect on stillbirth was observed among studies at low risk of bias (two RCTs; n = 3293; RR 0.97, 95% CI 0.52–1.82; I^2^ = 0%) or non-low risk (two RCTs; n = 558; RR 0.40, 95% CI 0.00–44.57; I^2^ = 0%). Although the between-subgroup difference was statistically significant (*p* = 0.018), the non-low-risk estimate was extremely imprecise and should be interpreted cautiously (Figure S24).

**Miscarriage.** No significant effect on miscarriage was observed in the single study at low risk of bias (n = 1391; RR 0.74, 95% CI 0.33–1.67) or among studies at non-low risk (two RCTs; n = 320; RR 0.90, 95% CI 0.01–94.58; I^2^ = 2.8%). The between-subgroup difference was not statistically significant (*p* = 0.71) (Figure S25).

### 3.4. Quality Assessment

**Figure 8:**
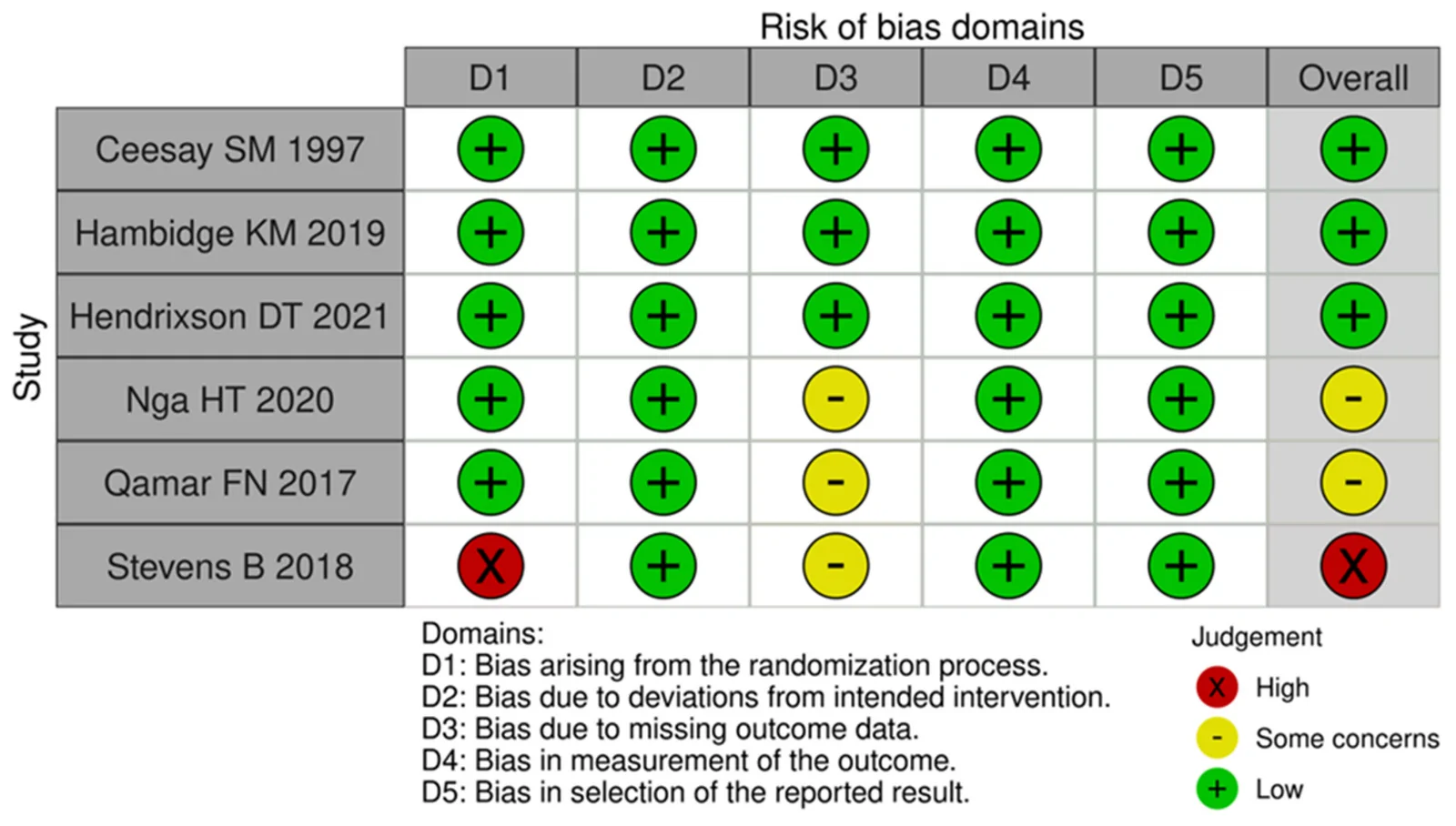
Risk of bias assessment summary [17,18,19,20,21,22].

Among the six included RCTs, three were assessed as having low risk of bias, two as having some concerns for bias, and one as having a high risk for bias based on the RoB2 tool. The detailed evaluation is presented in Figure 8. The funnel plots and trim-and-fill analyses were therefore considered exploratory, and no conclusion regarding the presence or absence of publication bias was drawn (Figures S26–S31).

## 4. Discussion

This systematic review and meta-analysis of six RCTs [17,18,19,20,21,22] found that nutritional interventions during pregnancy produced modest improvements in neonatal anthropometry. Nutritional interventions increased mean birth weight by 51.49 g and birth length by 0.29 cm compared with control conditions. The prediction intervals for both outcomes excluded the null, although LOO analyses indicated that statistical significance depended partly on the larger trials. No statistically significant effects were identified for head circumference, low birth weight, stillbirth, or miscarriage. The evidence for low birth weight and miscarriage was particularly imprecise, meaning that clinically relevant benefit or harm cannot be excluded.

The pooled estimates should not, however, be interpreted uniformly as the isolated effects of nutritional supplementation. Intervention composition, timing, nutritional content, comparator conditions, and accompanying care differed across trials. In particular, Hendrixson et al. [22]. evaluated a multicomponent package combining supplementary food with enhanced malaria prophylaxis, azithromycin, and testing and treatment for vaginal dysbiosis. Consequently, estimates incorporating this study represent the effect of the intervention package as implemented and cannot be attributed exclusively to its nutritional component.

The March 2026 meta-analysis by X S. et al. included the same six RCTs as the present review because no additional eligible randomized trials were identified by our updated search [7]. The added value of the present study therefore lies in independent data verification and methodological re-evaluation rather than in the inclusion of new trials. The previous review reported a statistically significant reduction in neonatal head circumference (MD −0.52 cm, 95% CI −0.63 to −0.42). In its forest plot, the intervention-group mean from Hambidge et al. appears to have been entered as 32.24 cm, whereas the original Women First report provides a mean of 33.24 ± 1.42 cm among 832 participants, compared with 33.18 ± 1.49 cm among 806 control participants [13]. Using the values reported in the original trial, our analysis found no statistically significant overall effect on head circumference (MD −0.14 cm, 95% CI −0.62 to 0.34). Because Hambidge et al. was one of the largest contributing trials, this one-centimetre discrepancy materially affects the pooled interpretation.

Model selection also influenced the interpretation of low birth weight. The previous review reported a statistically significant reduction (RR 0.75, 95% CI 0.64–0.87), whereas our random-effects analysis produced a similar point estimate but substantially greater uncertainty (RR 0.76, 95% CI 0.35–1.68). Although the previous review described random-effects modelling in its Methods, its principal summary table reported fixed-effect estimates for most relevant outcomes. Given the considerable clinical variability in population nutritional status, intervention composition and timing, comparator type, and accompanying co-interventions, we applied REML random-effects models consistently. These differences demonstrate that the interpretation of the existing evidence is sensitive to data extraction and statistical modelling assumptions.

Accordingly, the present study should be viewed as an independent methodological re-analysis of the same randomized evidence. Its contribution is the verification of trial-level data and a more extensive evaluation of heterogeneity, prediction intervals, risk of bias, subgroup interactions, leave-one-out robustness, and study influence. It does not represent an update based on the identification of additional randomized trials.

The improvements in birth weight and length are broadly consistent with previous systematic reviews of balanced energy–protein supplementation [3,4,6,18,19,20,21]. Imdad and Bhutta reported an average increase of approximately 73 g in birth weight, with potentially greater effects among undernourished women [17]. The somewhat smaller increase observed in the present analysis may reflect differences in eligibility criteria, nutritional formulations, intervention timing, adherence, background diets, and comparator conditions. Although the pooled increases in birth weight and birth length were statistically significant, their absolute magnitudes were small, and their clinical importance for an individual newborn remains uncertain. These findings should therefore not be interpreted as demonstrating a clinically meaningful improvement in neonatal health. Small shifts in neonatal anthropometry might still have population-level relevance in settings with a high prevalence of maternal undernutrition and fetal growth restriction; however, the present analysis did not demonstrate a statistically significant reduction in low birth weight or other adverse pregnancy outcomes.

Nutritional interventions did not significantly reduce the incidence of low birth weight in the present analysis. Although the pooled point estimate suggested a possible 24% risk reduction, the confidence interval was wide and crossed the null. This differs from some previous meta-analyses reporting significant reductions in low birth weight [3,6,7]. The discrepancy is likely related to the inclusion of only three eligible RCTs, substantial heterogeneity, and the application of a random-effects model, which appropriately produced greater uncertainty. Consequently, this result should not be interpreted as evidence that nutritional interventions have no effect on low birth weight. Rather, the available evidence was insufficient to establish a reliable effect. This distinction is important because low birth weight remains strongly associated with neonatal mortality, impaired childhood growth, and adverse health outcomes later in life [21,23,24,25].

The subgroup analyses provided only hypothesis-generating findings. Although statistically significant increases in birth weight and length were observed within trials targeting women with undernutrition, statistical significance in one subgroup and its absence in another do not establish a difference between subgroups. The formal tests for interaction were not statistically significant; therefore, the present evidence does not demonstrate that maternal undernutrition eligibility modified the effect of the interventions. Nevertheless, the pattern is biologically plausible and consistent with evidence suggesting that supplementation produces greater benefits when baseline energy or nutrient deficiencies are present [2,17,18,26,27]. Moreover, maternal undernutrition eligibility largely overlapped with comparator type in the included trials. The effects of population selection and comparator intensity therefore cannot be separated reliably.

A similar caution applies to the comparator-based analyses. Improvements in birth weight and length were statistically significant within studies using active comparators but not within those using inactive comparators; however, the tests for interaction were nonsignificant. There is therefore no evidence that comparator type modified the intervention effect.

No significant overall effect was observed for head circumference, and heterogeneity was substantial. The Baujat and LOO analyses identified Nga et al. [21] as the principal contributor to this heterogeneity. Importantly, the apparent reduction in head circumference was confined to studies at non-low risk of bias, whereas low-risk trials showed no significant effect. Although the risk-of-bias subgroup interaction was statistically significant, it was based on only two studies per subgroup and should be considered exploratory. Overall, the findings do not provide convincing evidence that nutritional interventions adversely affect neonatal head circumference.

Stillbirth and miscarriage were not significantly affected by nutritional interventions. The stillbirth estimate was consistent across studies and LOO analyses, with no detected heterogeneity. In contrast, the miscarriage estimate was highly imprecise because of the small number of events. These findings broadly correspond to previous reviews that have found inconsistent or uncertain effects of prenatal nutritional supplementation on fetal and perinatal mortality [3,6,19]. Larger trials are needed because even substantial studies may lack adequate statistical power for uncommon outcomes such as stillbirth and miscarriage.

The results of the sensitivity analyses add important context. For birth weight and length, all LOO estimates remained in the direction of benefit, but significance was lost after removing certain large trials. Thus, the direction of effect was generally consistent, while its precision depended on the most informative studies. Differences in baseline nutritional status, dietary context, supplement composition, commencement of supplementation, adherence, infection-control measures, and access to antenatal care may explain the observed between-study variability [16,17,18,19,20,21].

The variation in supplement formulation and energy content, timing of initiation, comparator intensity, background antenatal care, and accompanying co-interventions provides a plausible clinical explanation for the moderate-to-substantial statistical heterogeneity observed for birth weight, head circumference, and low birth weight. Nevertheless, because only a few trials contributed to each analysis and several characteristics differed simultaneously, the available data could not reliably identify any single source of heterogeneity.

**Figure 9:**
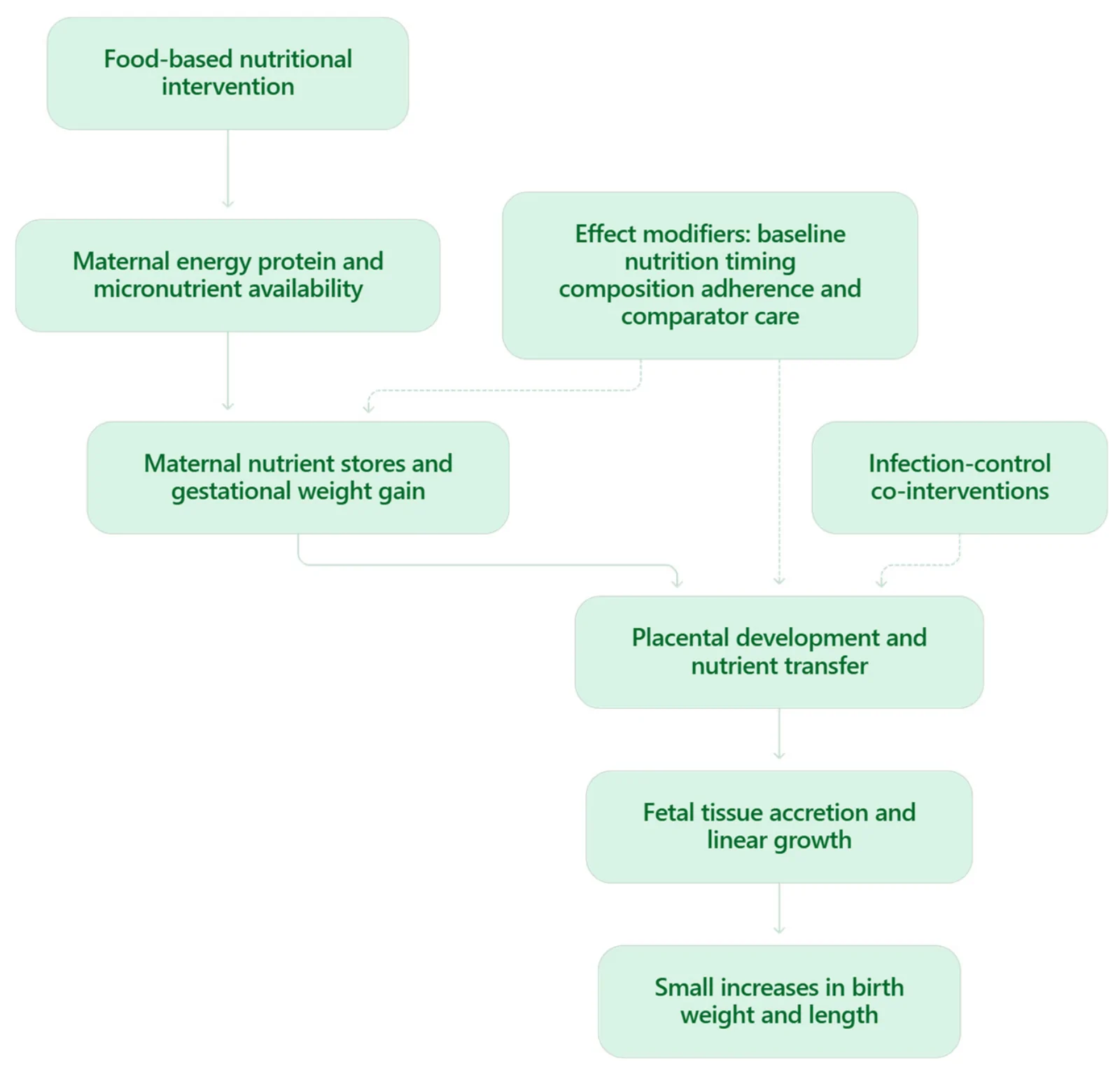
Proposed biological and contextual pathways linking food-based nutritional interventions during pregnancy to maternal and neonatal outcomes. Increased maternal energy, protein, and micronutrient availability may support maternal nutritional status, placental development, nutrient transfer, and fetal growth. Baseline maternal nutritional status, intervention timing and composition, adherence, background diet, infection and inflammation, comparator care, and accompanying co-interventions may modify these pathways. The framework presents biologically plausible mechanisms and should not be interpreted as a causal model validated directly by the included trials.

The potential biological and contextual pathways underlying the observed findings are summarized in Figure 9. Food-based interventions may support fetal growth by increasing maternal energy, protein, and micronutrient availability, improving maternal nutrient stores and gestational weight gain, and supporting placental development and nutrient transfer. These pathways may promote fetal tissue accretion and linear growth. However, the magnitude of any effect may depend on baseline maternal nutritional status, background diet, intervention timing and composition, adherence, infection and inflammation, and comparator care. In multicomponent interventions, infection-control measures may also influence maternal inflammation and placental function independently of nutritional supplementation [1,18,20] (Figure 9).

This review has several strengths, including restriction to randomized trials, application of random-effects models, reporting of prediction intervals, RoB 2 assessment, and the use of LOO and Baujat analyses. Only three to five trials contributed to each outcome. Consequently, the subgroup interaction tests, risk-of-bias stratifications, influence analyses, and assessments of small-study effects had limited power and may yield unstable findings. All secondary analyses should therefore be considered exploratory. The interventions differed in composition, dose, duration, timing, and associated co-interventions, while the control groups ranged from usual care to active nutritional comparators. Some trials also commenced supplementation before conception or combined nutritional supplementation with infection-control measures, introducing clinical indirectness. The subgroup analyses were based on study-level characteristics, were not independent of one another, and may be affected by ecological confounding. Definitions and gestational-age thresholds for miscarriage and stillbirth were not fully standardized or consistently reported across the included trials, which may have introduced additional clinical heterogeneity into these pooled outcomes. Furthermore, restriction to English-language and peer-reviewed publications may have introduced language and publication bias and may have excluded relevant unpublished studies or studies reporting null or unfavourable findings. The review may therefore not represent the complete body of available evidence.

## 5. Conclusions

Food-based nutritional intervention strategies were associated with small increases in birth weight and birth length, but the clinical importance of these differences remains uncertain, and effects from multicomponent interventions cannot necessarily be attributed solely to nutritional supplementation. Although greater benefits were observed in trials targeting undernourished women, subgroup differences were not statistically significant. Further adequately powered RCTs are needed to identify the populations most likely to benefit and determine the optimal timing and composition of supplementation.

## Figures and Tables

**Table 1:** Baseline characteristics of the included studies.

Study, Setting & Design	Population/ Key Eligibility	Intervention	Control	Sample Size	Age, Sex & Nutritional Profile	Timing/ Follow-Up	Primary Outcomes
Ceesay et al., 1997 [17] Country: The Gambia Design: 5-year cluster RCT; 28 rural villages	Population: Chronically undernourished rural pregnant women; twin births excluded from anthropometric analyses. Recruitment: All women aged 15–45 years in participating villages.	Prenatal supplement: Two high-energy groundnut biscuits/day (4250 kJ; 22 g protein) from about 20 weeks’ gestation to delivery.	Delayed supplement: Same biscuits provided for 20 weeks after delivery; no prenatal food supplement. Co-interventions: Routine antenatal care, iron-folate, tetanus and malaria prophylaxis in both groups.	Analysis sample: 2047 births (I: 1010; C: 1037).	Age, mean +/− SD: I 24.0 +/− 6.2; C 23.7 +/− 6.4 y. Female: 100%. Nutrition: Chronically undernourished population; no individual BMI entry cut-off.	Exposure: Mean 82 +/− 31 prenatal supplement days. Assessments: Birth measures < 48 h; mortality through day 365.	Primary: Birth weight and infant survival. Other: LBW, length, head circumference, gestation and perinatal mortality.
Hambidge et al., 2019 [19] (Women First) Country: DRC, Guatemala, India and Pakistan Design: 3-arm, RCT, nonmasked multisite efficacy trial	Population: Women aged 16–35 y, parity 0–5, expecting conception within 18 months. Selection: Not restricted by BMI; severe anemia was treated before enrollment.	Arm 1: Daily lipid-based micronutrient supplement (118 kcal; 2.6 g protein) from >=3 months preconception to delivery. Arm 2: Same supplement from late first trimester to delivery. Additional: 300-kcal protein–energy supplement if BMI < 20 or gestational weight gain was low.	Arm 3: No trial nutrition supplement; usual locally prescribed/self-administered supplements permitted.	Analysis sample: 2162 pregnancies (I: 1094; C: 1068).	Mean age: NR; eligible range 16–35 y. Female: 100%. BMI < 18.5: Arm 1 23.6%; Arm 2 23.5%; C 22.1% (primary-outcome cohort).	Period: Preconception enrollment through delivery. Newborn assessment: Anthropometry within 48 h.	Primary: Newborn length-for-age z score (LAZ). Other: Weight, head circumference, BMI, LBW, SGA and preterm birth.
Hendrixson et al., 2021 [22] Country: Sierra Leone Design: 2-arm, RCT; masked birth assessors	Population: Undernourished pregnant women attending 43 antenatal clinics. Eligibility: MUAC <= 23 cm and fundal height < 35 cm.	Package: RUSF (520 kcal; 18 g protein) plus enhanced malaria prophylaxis, two azithromycin doses, and bacterial-vaginosis testing/treatment.	Standard care: WFP corn-soy blend plus oil (589 kcal; 17.5 g protein), iron-folate and standard malaria prophylaxis. Both groups: Bednet and albendazole.	Analysis sample: 1391 pregnancies (I: 710; C: 681).	Age, mean +/− SD: I 21.0 +/− 4.7; C 21.5 +/− 4.9 y. Female: 100%. MUAC, mean +/− SD: I 22.3 +/− 0.7; C 22.3 +/− 0.8 cm.	Exposure: Enrollment to delivery; median participation I 15.4, C 14.9 weeks. Infant visits: Birth, 6 weeks, 3 and 6 months.	Primary: Newborn length. Other: Birth weight/head circumference, maternal growth, infant growth and survival to 6 months.
Nga et al., 2020 [21] (VINAVAC) Country: Vietnam Design: 3-arm, RCT	Population: Nulliparous women aged 18–30 y planning pregnancy within 1 year. Eligibility: Nonpregnant at entry; BMI >= 17 kg/m^2^; able to attend weekday feeding.	PC-T: 190-kcal nutrient-rich local-food supplement 5 d/week from preconception to term. MG-T: Same supplement from mid-gestation to term.	RPC: Routine prenatal care/nutrition counseling; no study food supplement.	Analysis sample: 310 women (I: 153; C: 157).	Age, mean +/− SD: 21.5 +/− 3.2/21.1 +/− 2.7/21.6 +/− 2.6 y. Female: 100%. BMI, mean +/− SD: 19.6 +/− 1.6/19.7 +/− 1.8/19.8 +/− 1.9 kg/m^2^ (completers).	Period: Preconception through delivery. Birth measures: Hospital at delivery; study team within 7 days.	Primary: Birth weight, length and prematurity. Other: Maternal weight gain and nutritional biomarkers.
Qamar, 2017 [18] Country: Pakistan Design: 2-arm, RCT, open-label trial	Population: Undernourished pregnant women in two low-income peri-urban Karachi communities. Eligibility: BMI <= 19.9 kg/m^2^; identified before 14 gestational weeks.	Daily supplement: 90-g energy-dense micronutrient biscuits (450 kcal/day) from 14 weeks’ gestation to delivery.	Food ration: Wheat flour (5 kg) and oil (1 L) distributed every 2 weeks.	Analysis sample: 248 pregnancies (I: 125; C: 123).	Age, mean +/− SD: I 23.4 +/− 4.7; C 23.0 +/− 4.5 y. Female: 100%. BMI, mean +/− SD: I 18.4 +/− 1.47; C 18.2 +/− 1.46 kg/m^2^.	Enrollment: 10.6 +/− 1.7 gestational weeks. Exposure: 14 weeks to delivery. Newborn assessment: Within 48 h.	Primary: Neonatal birth weight. Other: Maternal hemoglobin and ferritin.
Stevens et al., 2018 [20] Country: Bangladesh Design: RCT; control villages purposively matched	Population: Undernourished pregnant women in 12 rural villages. Eligibility: MUAC <= 22.1 cm; singleton pregnancy; no need for medical referral.	Daily supplement: One serving of locally produced balanced protein–energy food from enrollment to term; intake closely observed.	Usual services: Same nutrition screening, education, antenatal/postnatal services and iron–folic acid; no food supplement.	Analysis sample: 87 pregnancies (I: 58; C: 29).	Mean age: NR. Median age (IQR): I 21 (10); C 22.5 (7) y (available n = 31/18). Female: 100%. Nutrition: MUAC <= 22.1 cm; 52.6% were <148 cm tall overall.	Exposure: Enrollment during pregnancy to term. Infant visits: Birth, 1, 3 and 6 months.	Primary: Birth weight and infant weight/MUAC at 1, 3 and 6 months. Other: Preterm birth, pregnancy loss and infant mortality.

Notes. Values are mean +/− SD unless otherwise stated. Female participation was 100% in all trials because the study populations comprised women who were pregnant or planning pregnancy. The Sample size column reports the largest outcome-specific denominator used in this meta-analysis for each trial; denominators varied by outcome. Across the six trials, 6245 participants contributed to at least one analysis (I 3150; C 3095). C = control; I = intervention; NR = not reported. Abbreviations. BMI, body mass index; DRC, Democratic Republic of the Congo; LAZ, length-for-age z score; LBW, low birth weight; MG-T, mid-gestation-to-term; MUAC, mid-upper arm circumference; PC-T, preconception-to-term; RCT, randomized controlled trial; RPC, routine prenatal care; RUSF, ready-to-use supplementary food; SGA, small for gestational age; WFP, World Food Programme. Contribution to quantitative syntheses. Birth weight: Hambidge et al., Hendrixson et al., Nga et al., Qamar, and Stevens et al.; birth length and head circumference: Hambidge et al., Hendrixson et al., Nga et al., and Qamar; low birth weight: Ceesay et al., Hambidge et al., and Nga et al.; stillbirth: Hambidge et al., Hendrixson et al., Nga et al., and Qamar; miscarriage: Hendrixson et al., Qamar, and Stevens et al.

## Data Availability

All data are publicly available from the included studies.

## Supplementary Materials

The following supporting information can be downloaded at: https://www.mdpi.com/article/10.3390/jcm15187297/s1, Figure S1: LOO sensitivity analysis of birth weight showing consistently positive effect estimates after the sequential omission of individual studies (MD range 36.18–60.64 g); omitting Nga et al. (2020) [21] eliminated between-study heterogeneity (I^2^ = 0%). Figure S2: Baujat plot for birth weight identifying Nga et al. (2020) [21] as the largest contributor to heterogeneity and the most influential study, with Hendrixson et al. (2021) [22] also showing substantial influence on the pooled estimate. Figure S3: LOO sensitivity analysis of birth length showing consistently positive effect estimates (MD range 0.10–0.33 cm); statistical significance was lost after omitting Hambidge et al. (2019) [19] or Hendrixson et al. (2021) [22], while omission of Nga et al. (2020) [21] eliminated heterogeneity (I^2^ = 0%). Figure S4: Baujat plot for birth length identifying Nga et al. (2020) [21] as the largest contributor to heterogeneity and Hambidge et al. (2019) [19] as the most influential study on the pooled estimate. Figure S5: LOO sensitivity analysis of birth head circumference showing no significant effect in any iteration; omission of Nga et al. (2020) [21] eliminated heterogeneity, identifying it as the principal source of between-study variability. Figure S6: Baujat plot for birth head circumference identifying Nga et al. (2020) [21] as the largest contributor to heterogeneity and the most influential study, while Hendrixson et al. (2021) [22] showed a smaller influence on the pooled estimate. Figure S7: LOO sensitivity analysis of low birth weight showing no significant effect in any iteration, with persistent substantial heterogeneity and marked imprecision after omitting Ceesay et al. (1997) [17] or Hambidge et al. (2019) [19]. Figure S8: Baujat plot for low birth weight identifying Nga et al. (2020) [21] as the largest contributor to heterogeneity and Ceesay et al. (1997) [17] as the most influential study on the pooled estimate. Figure S9: LOO sensitivity analysis of still birth showing no significant effect in any iteration and no observed between-study heterogeneity. Figure S10: LOO sensitivity analysis of miscarriage showing no significant effect in any iteration, with negligible heterogeneity and marked imprecision after omitting Hendrixson et al. (2021) [22] or Qamar (2017) [18]. Figure S11: Subgroup forest plot of birth weight according to maternal undernutrition eligibility, showing a significant increase among targeted populations but no significant effect among non-targeted populations; the between-subgroup difference was not statistically significant. Figure S12: Subgroup forest plot of birth length according to maternal undernutrition eligibility, showing a significant increase among targeted populations but no significant effect among non-targeted populations; the between-subgroup difference was not statistically significant. Figure S13: Subgroup forest plot of birth head circumference according to maternal undernutrition eligibility, showing no significant effect in either targeted or non-targeted populations and no statistically significant between-subgroup difference. Figure S14: Subgroup forest plot of stillbirth according to maternal undernutrition eligibility, showing no significant effect in either targeted or non-targeted populations and no statistically significant between-subgroup difference. Figure S15: Subgroup forest plot of birth weight according to comparator type, showing a significant increase in trials with active comparators but no significant effect in trials with inactive comparators; the between-subgroup difference was not statistically significant. Figure S16: Subgroup forest plot of birth length according to comparator type, showing a significant increase in trials with active comparators but no significant effect in trials with inactive comparators; the between-subgroup difference was not statistically significant. Figure S17: Subgroup forest plot of birth head circumference according to comparator type, showing no significant effect in trials with either active or inactive comparators and no statistically significant between-subgroup difference. Figure S18: Subgroup forest plot of stillbirth according to comparator type, showing no significant effect in trials with either active or inactive comparators and no statistically significant between-subgroup difference. Figure S19: Subgroup forest plot of miscarriage according to comparator type, showing no significant effect in trials with active comparators or in the single trial with an inactive comparator, and no statistically significant between-subgroup difference. Figure S20: Sensitivity forest plot of birth weight stratified by overall risk of bias, showing a significant increase in low-risk studies but no significant effect in non-low-risk studies; the between-subgroup difference was not statistically significant. Figure S21: Sensitivity forest plot of birth length stratified by overall risk of bias, showing a significant increase in low-risk studies but no significant effect in non-low-risk studies; the between-subgroup difference did not reach statistical significance. Figure S22: Sensitivity forest plot of birth head circumference stratified by overall risk of bias, showing no significant effect in low-risk studies but a significant reduction in non-low-risk studies; the between-subgroup difference was statistically significant. Figure S23: Sensitivity forest plot of low birth weight stratified by overall risk of bias, showing no significant effect in low-risk studies or in the single non-low-risk study, and no statistically significant between-subgroup difference. Figure S24: Sensitivity forest plot of stillbirth stratified by overall risk of bias, showing no significant effect within either risk group; although the between-subgroup difference was statistically significant, the non-low-risk estimate was extremely imprecise and should be interpreted cautiously. Figure S25: Sensitivity forest plot of miscarriage stratified by overall risk of bias, showing no significant effect in the single low-risk study or among non-low-risk studies, and no statistically significant between-subgroup difference. Figure S26: Contour-enhanced funnel plot for birth weight. Although some asymmetry is visually apparent, publication bias cannot be assessed reliably because fewer than 10 studies were included. Figure S27: Contour-enhanced funnel plot for birth length. Visual asymmetry is apparent, but publication bias cannot be assessed reliably because only four studies were included. Figure S28: Contour-enhanced funnel plot for birth head circumference. Visual asymmetry is apparent, but publication bias cannot be assessed reliably because only four studies were included. Figure S29: Contour-enhanced funnel plot for low birth weight. Visual asymmetry is apparent, but publication bias cannot be assessed reliably because only three studies were included. Figure S30: Contour-enhanced funnel plot for stillbirth. Some visual asymmetry is apparent, but publication bias cannot be assessed reliably because only four studies were included. Figure S31: Contour-enhanced funnel plot for miscarriage. Visual asymmetry is apparent, but publication bias cannot be assessed reliably because only three studies were included. Figure S32: PRISMA checklist.
