# Supplementary material for: Effects of Nutritional Interventions During Pregnancy on Maternal and Neonatal Outcomes: A Systematic Review and Meta-Analysis of Randomized Controlled Trials
Source: J Clin Med. 2026 Sep 19;15(18):7297. doi: 10.3390/jcm15187297 (PMC13607848; doi:10.3390/jcm15187297)
Supplement: Supplementary file 1 [file jcm-15-07297-s001.zip › jcm-4553755-supplementary.pdf]

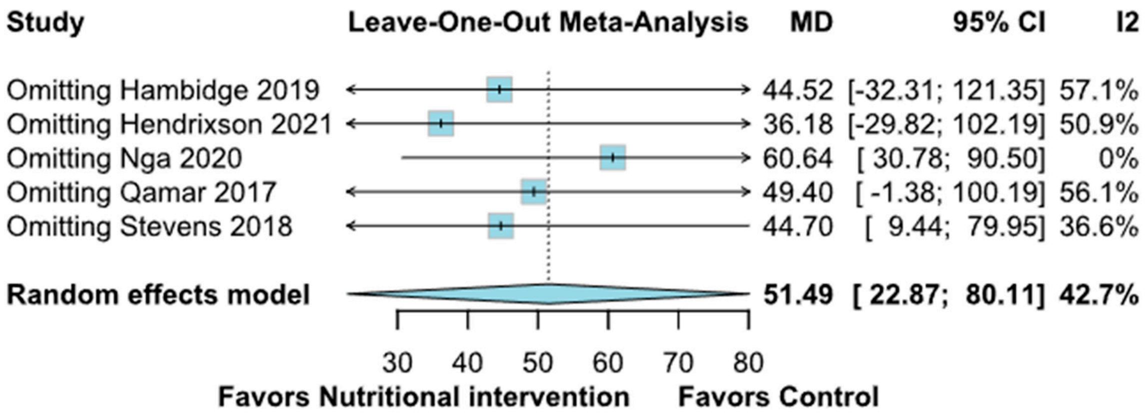

Figure S1. LOO sensitivity analysis of birth weight showing consistently positive effect estimates after the sequential omission of individual studies (MD range 36.18–60.64 g); omitting Nga et al. (2020) eliminated between-study heterogeneity ($I^2 = 0\%$).

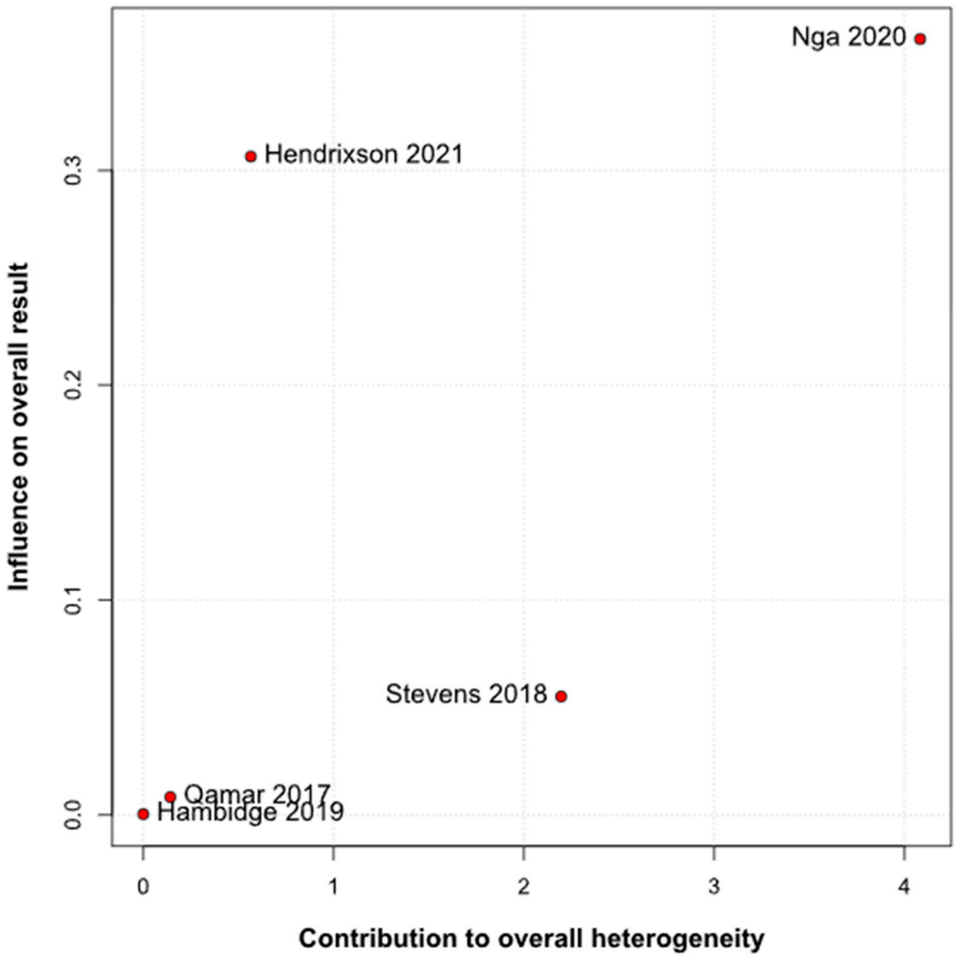

Figure S2. Baujat plot for birth weight identifying Nga et al. (2020) as the largest contributor to heterogeneity and the most influential study, with Hendrixson et al. (2021) also showing substantial influence on the pooled estimate.

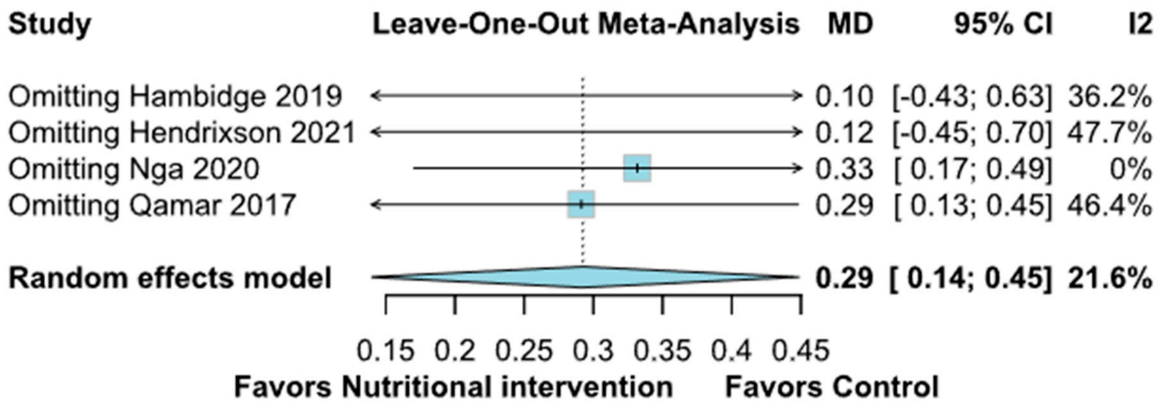

Figure S3. LOO sensitivity analysis of birth length showing consistently positive effect estimates (MD range 0.10–0.33 cm); statistical significance was lost after omitting Hambidge et al. (2019) or Hendrixson et al. (2021), while omission of Nga et al. (2020) eliminated heterogeneity ($I^2 = 0\%$).

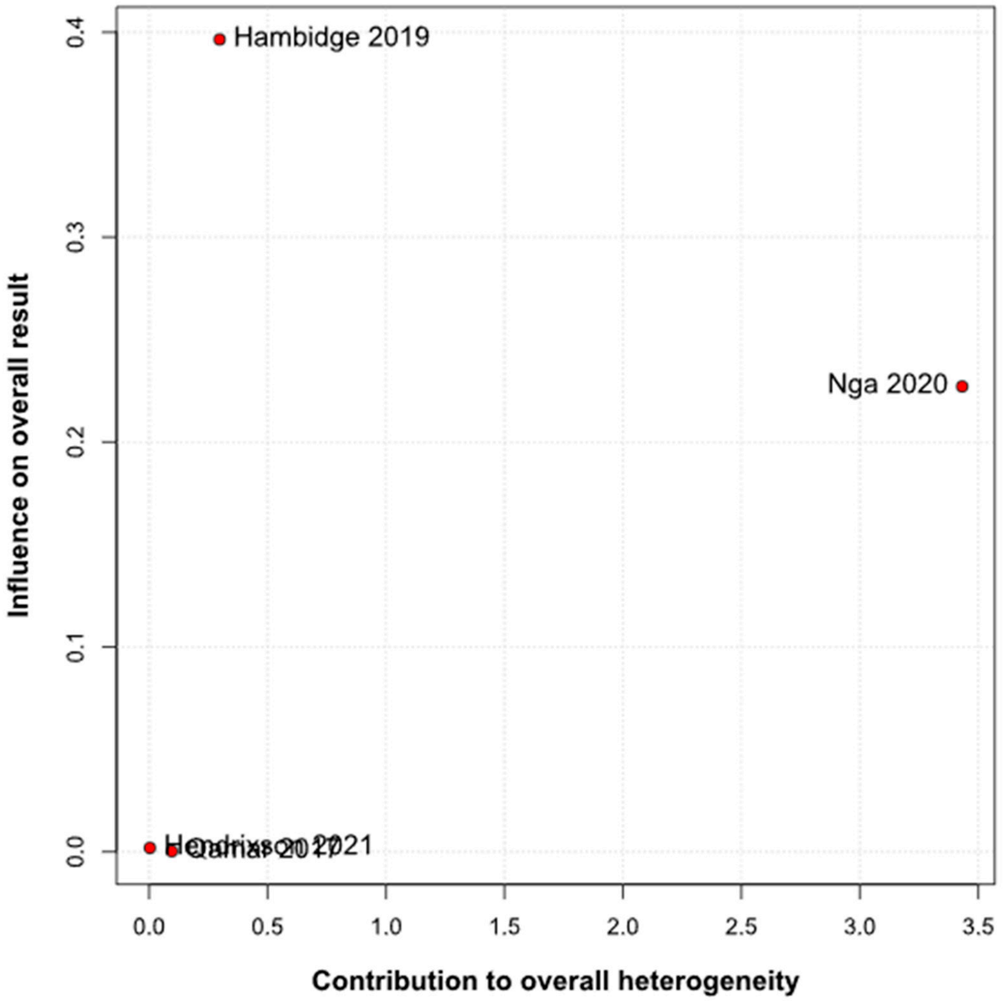

Figure S4. Baujat plot for birth length identifying Nga et al. (2020) as the largest contributor to heterogeneity and Hambidge et al. (2019) as the most influential study on the pooled estimate.

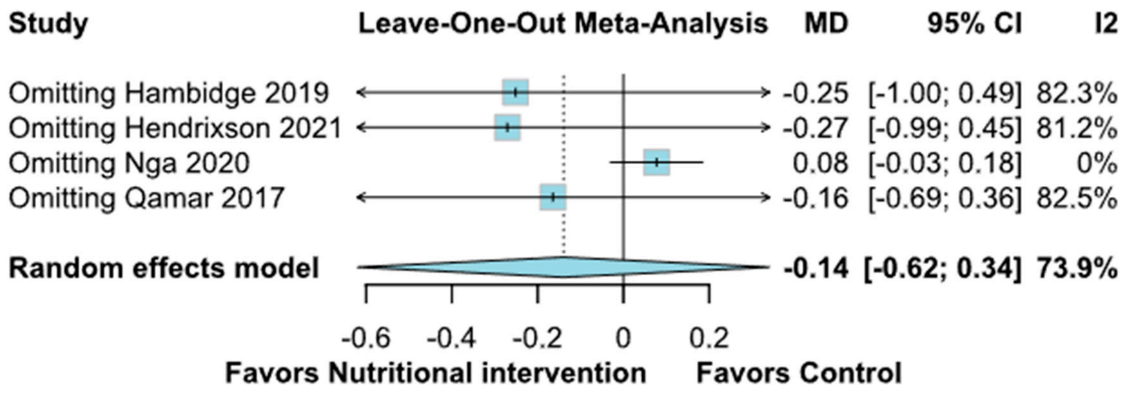

Figure S5. LOO sensitivity analysis of birth head circumference showing no significant effect in any iteration; omission of Nga et al. (2020) eliminated heterogeneity, identifying it as the principal source of between-study variability.

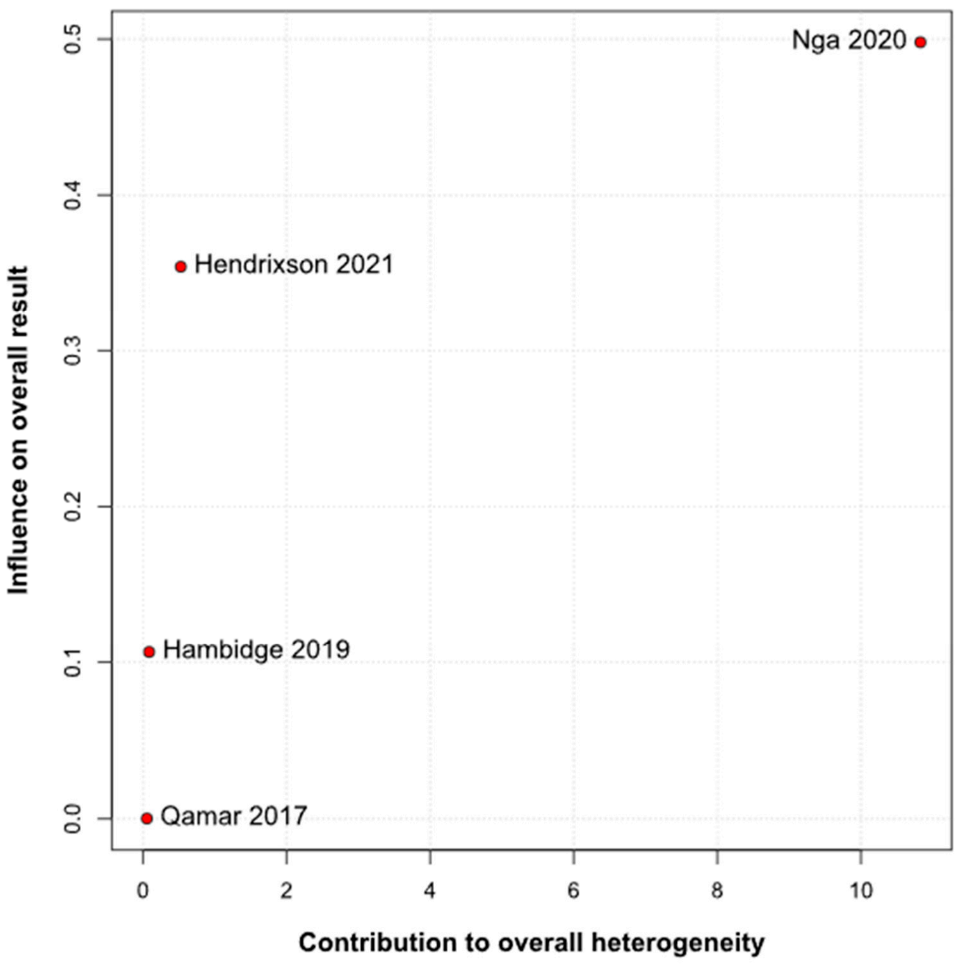

Figure S6. Baujat plot for birth head circumference identifying Nga et al. (2020) as the largest contributor to heterogeneity and the most influential study, while Hendrixson et al. (2021) showed a smaller influence on the pooled estimate.

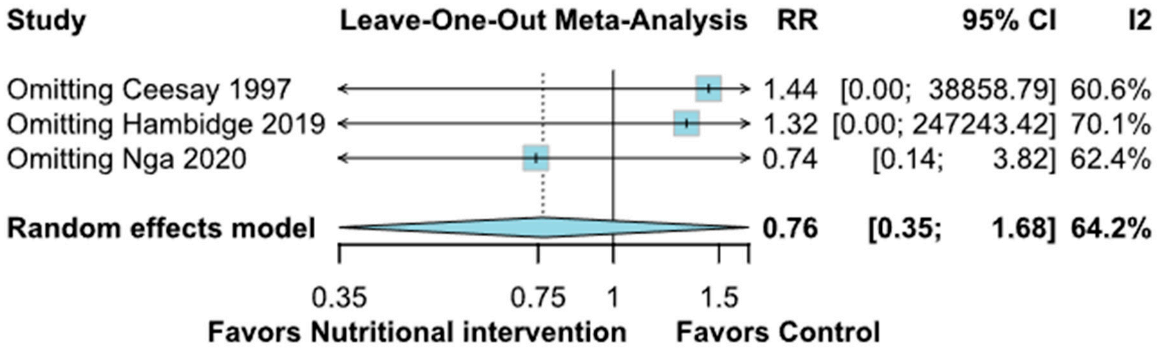

Figure S7. LOO sensitivity analysis of low birth weight showing no significant effect in any iteration, with persistent substantial heterogeneity and marked imprecision after omitting Ceesay et al. (1997) or Hambidge et al. (2019).

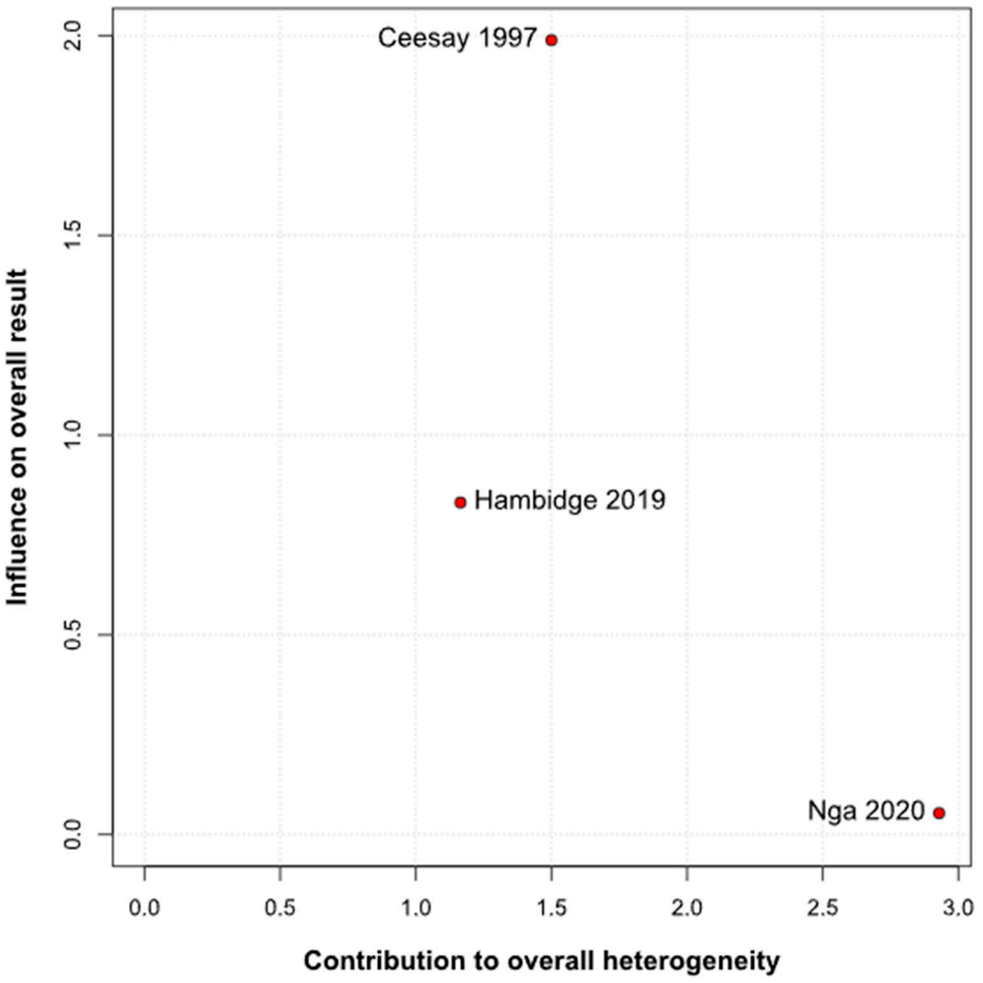

Figure S8. Baujat plot for low birth weight identifying Nga et al. (2020) as the largest contributor to heterogeneity and Ceesay et al. (1997) as the most influential study on the pooled estimate.

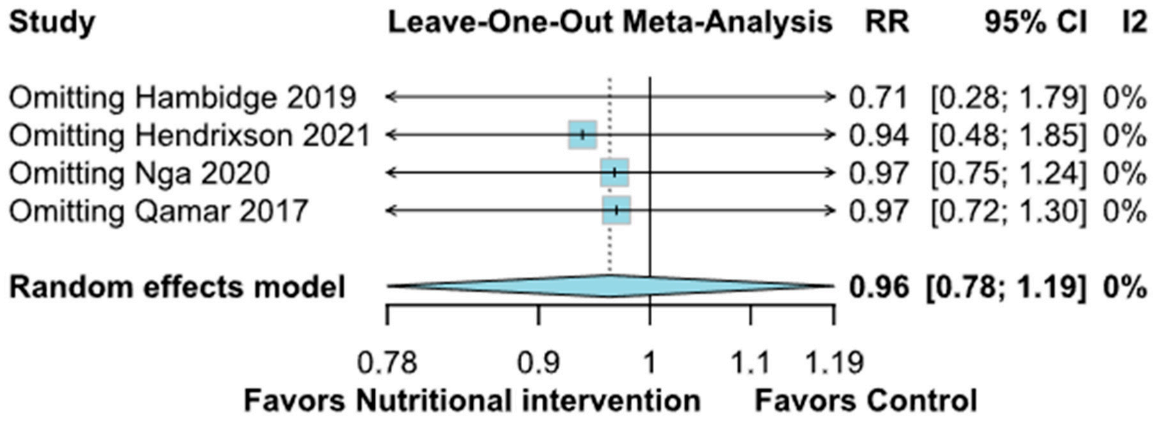

Figure S9. LOO sensitivity analysis of stillbirth showing no significant effect in any iteration and no observed between-study heterogeneity.

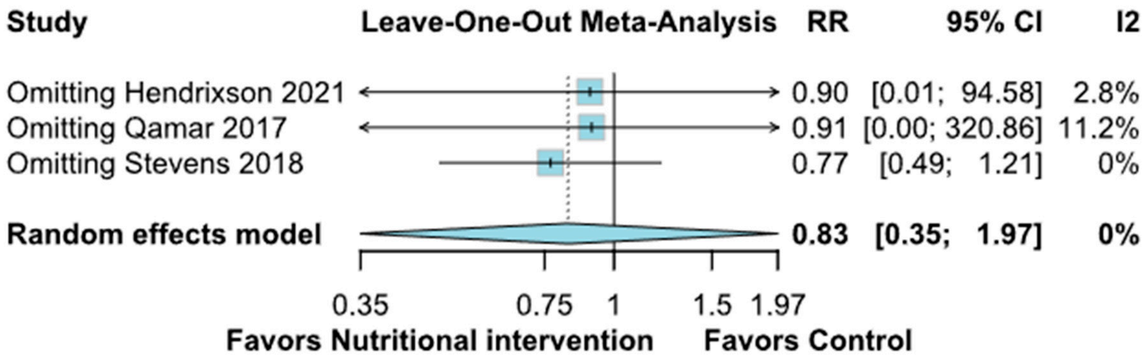

Figure S10. LOO sensitivity analysis of miscarriage showing no significant effect in any iteration, with negligible heterogeneity and marked imprecision after omitting Hendrixson et al. (2021) or Qamar et al. (2017).

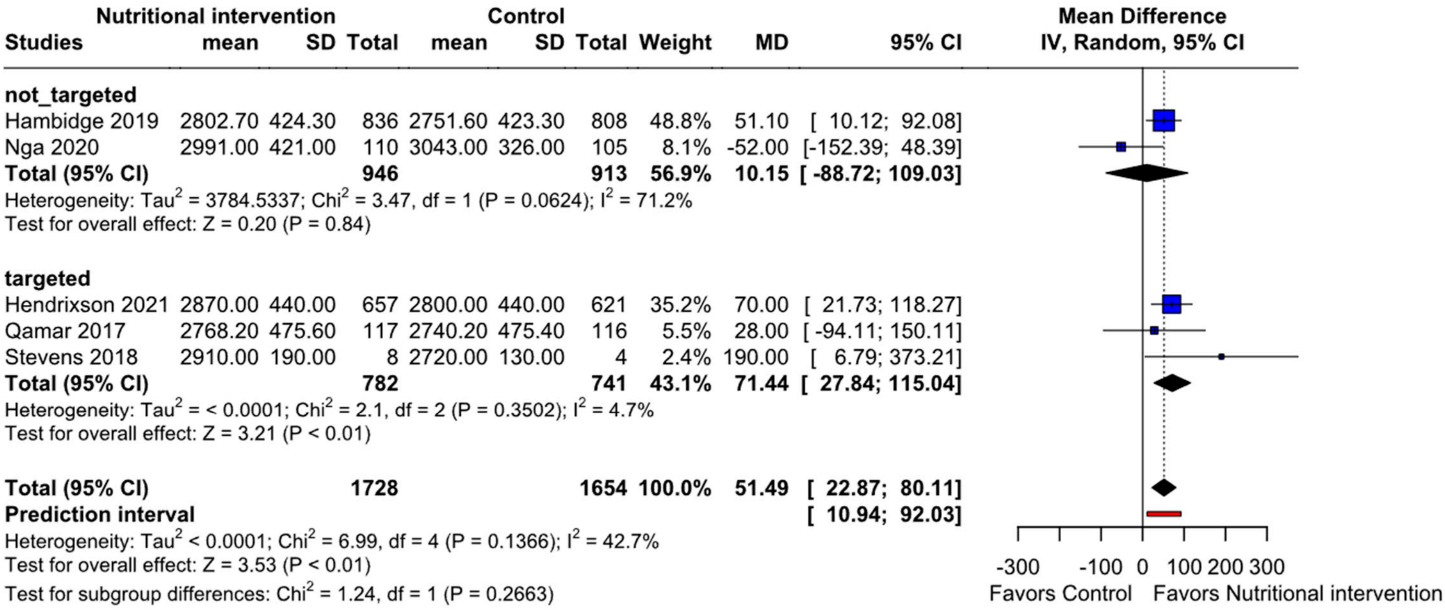

Figure S11. Subgroup forest plot of birth weight according to maternal undernutrition eligibility, showing a significant increase among targeted populations but no significant effect among non-targeted populations; the between-subgroup difference was not statistically significant.

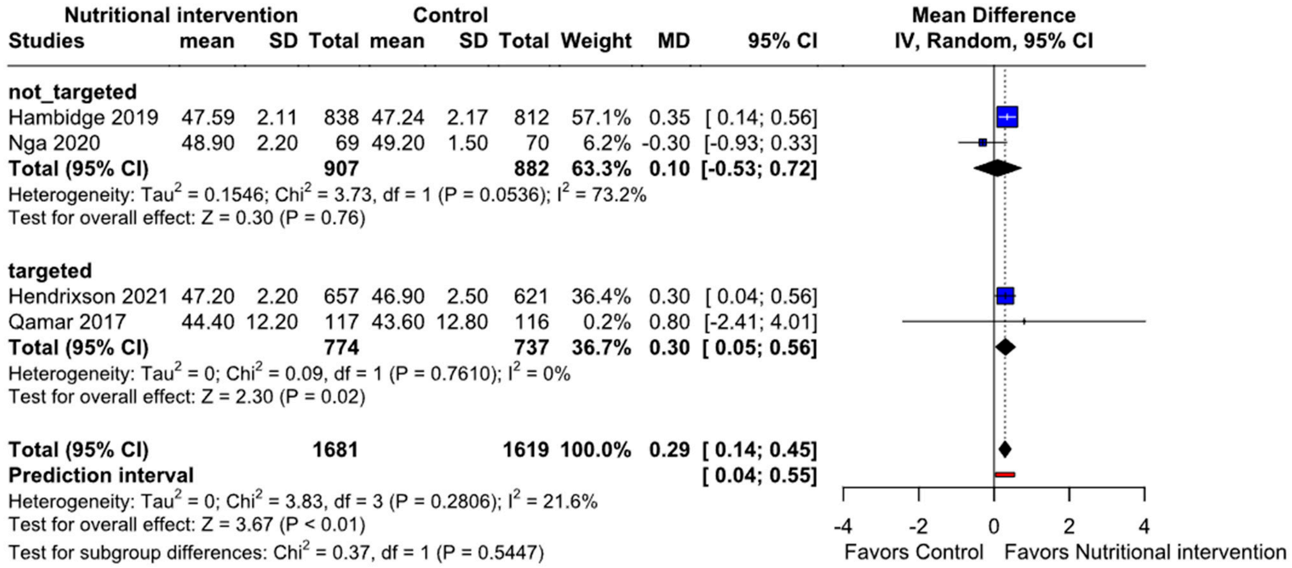

Figure S12. Subgroup forest plot of birth length according to maternal undernutrition eligibility, showing a significant increase among targeted populations but no significant effect among non-targeted populations; the between-subgroup difference was not statistically significant.

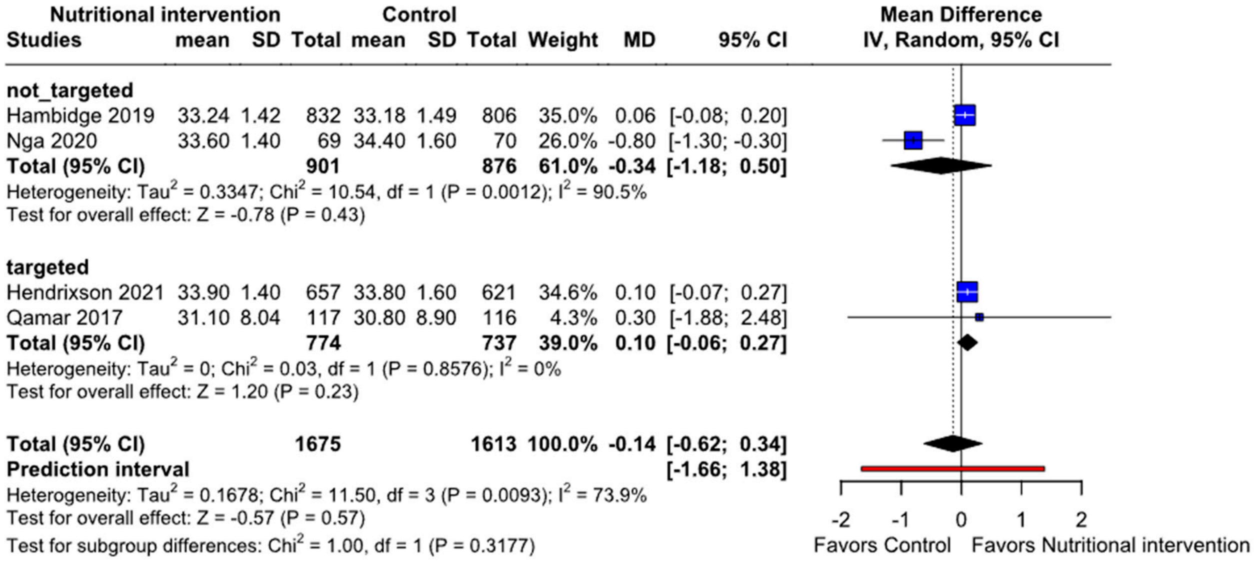

Figure S13. Subgroup forest plot of birth head circumference according to maternal undernutrition eligibility, showing no significant effect in either targeted or non-targeted populations and no statistically significant between-subgroup difference.

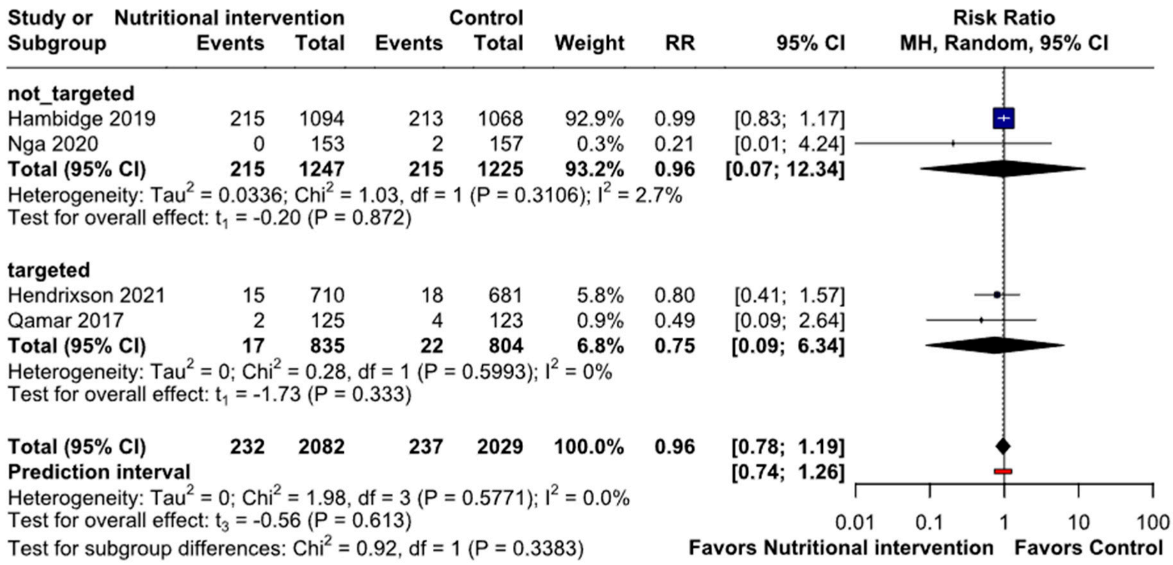

Figure S14. Subgroup forest plot of stillbirth according to maternal undernutrition eligibility, showing no significant effect in either targeted or non-targeted populations and no statistically significant between-subgroup difference.

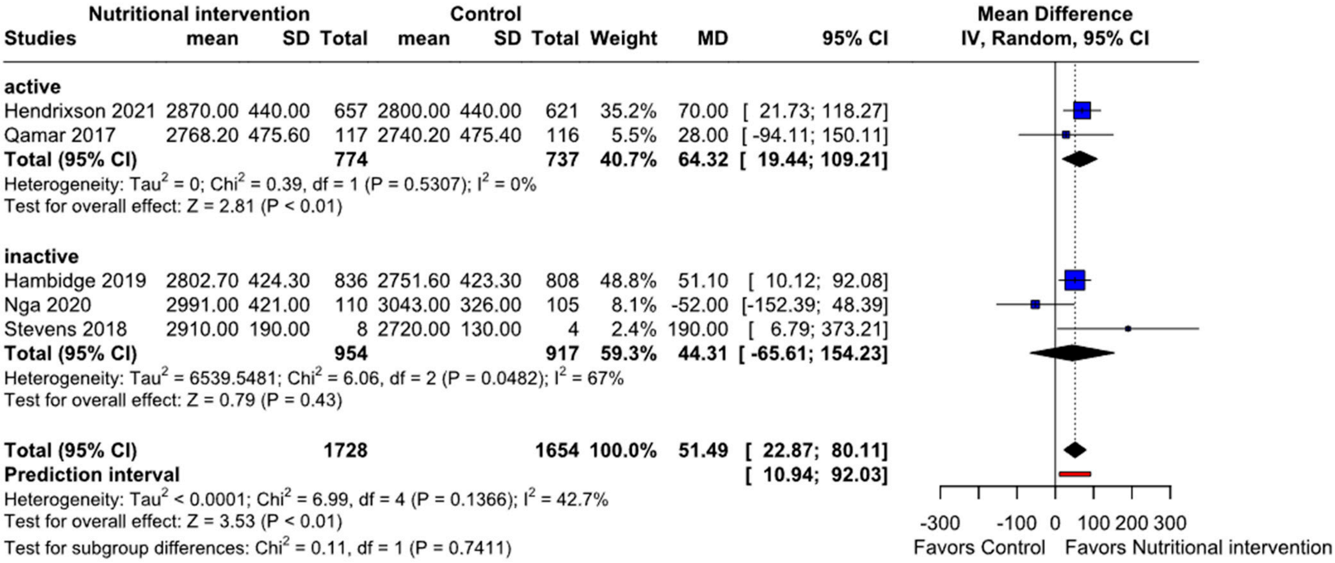

Figure S15. Subgroup forest plot of birth weight according to comparator type, showing a significant increase in trials with active comparators but no significant effect in trials with inactive comparators; the between-subgroup difference was not statistically significant

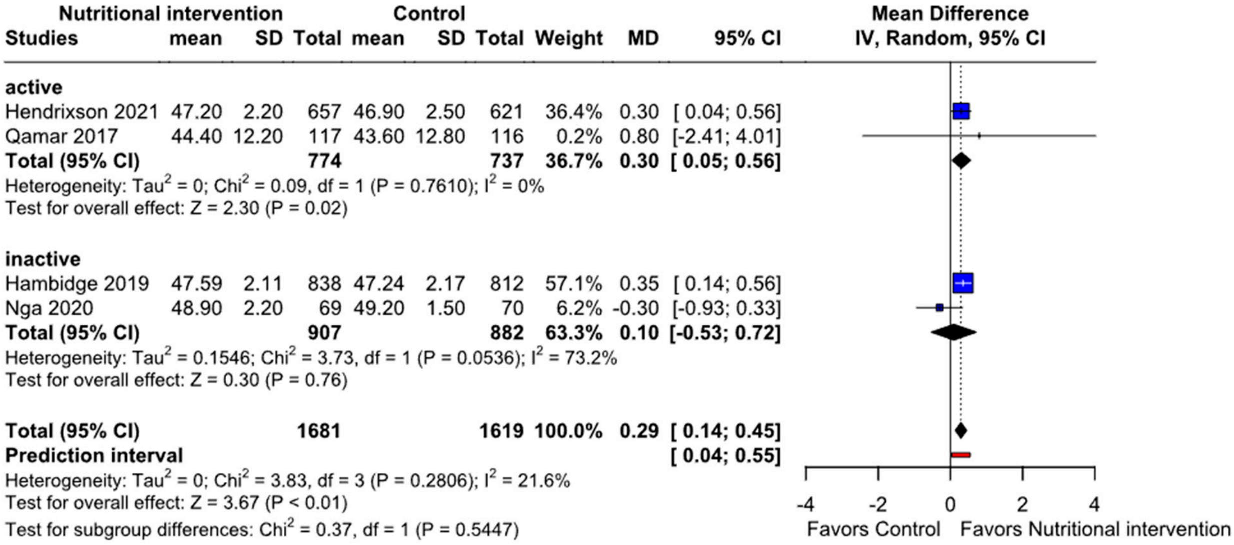

Figure S16. Subgroup forest plot of birth length according to comparator type, showing a significant increase in trials with active comparators but no significant effect in trials with inactive comparators; the between-subgroup difference was not statistically significant.

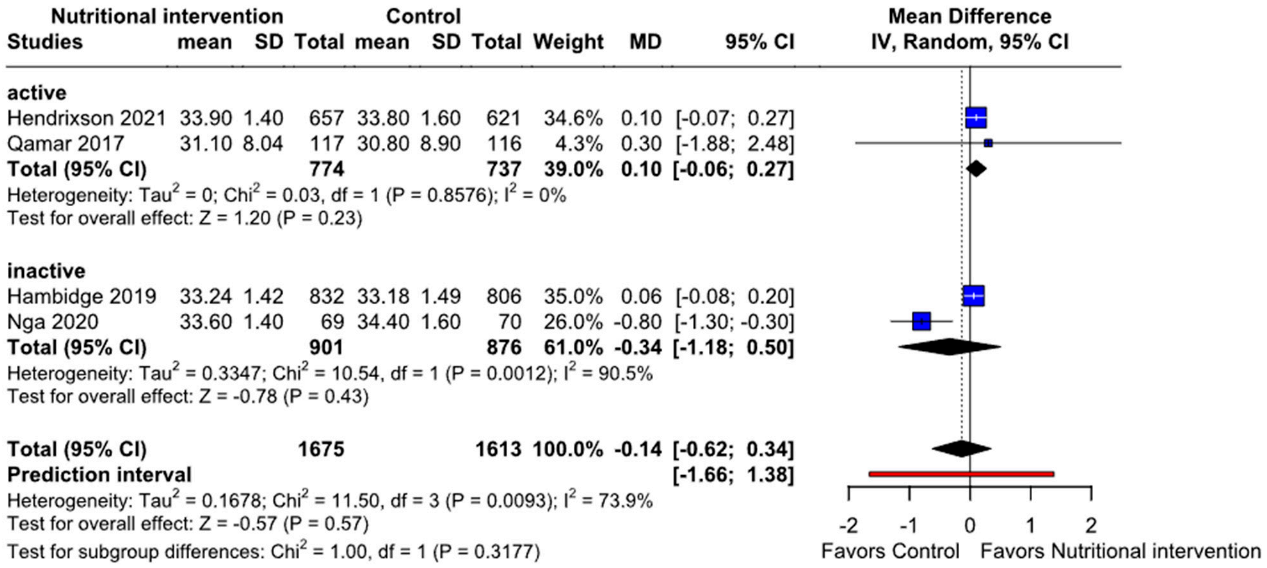

Figure S17. Subgroup forest plot of birth head circumference according to comparator type, showing no significant effect in trials with either active or inactive comparators and no statistically significant between-subgroup difference

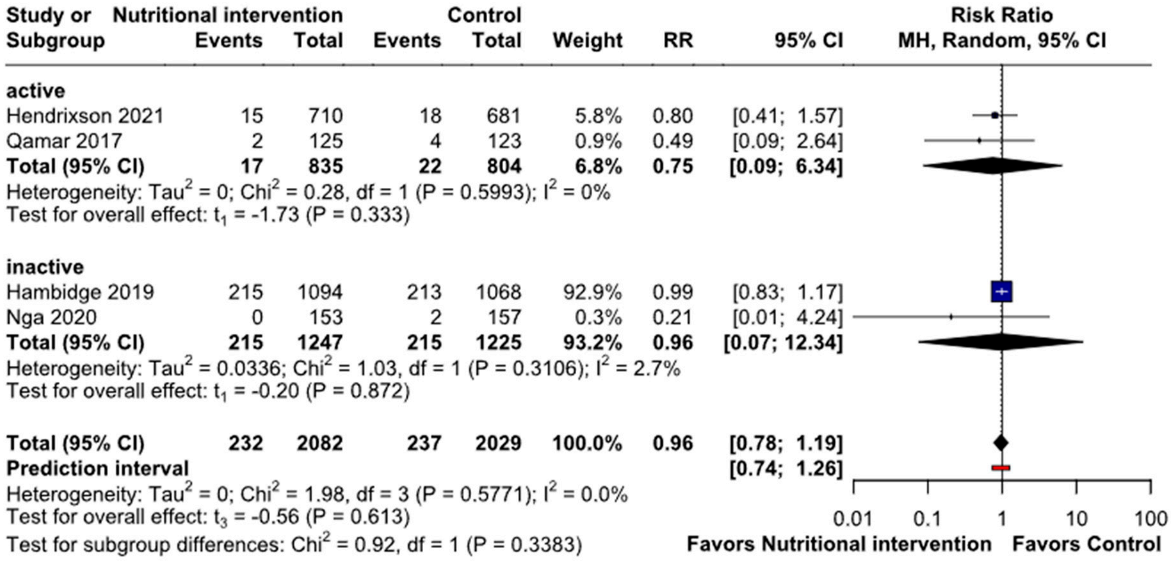

Figure S18. Subgroup forest plot of stillbirth according to comparator type, showing no significant effect in trials with either active or inactive comparators and no statistically significant between-subgroup difference.

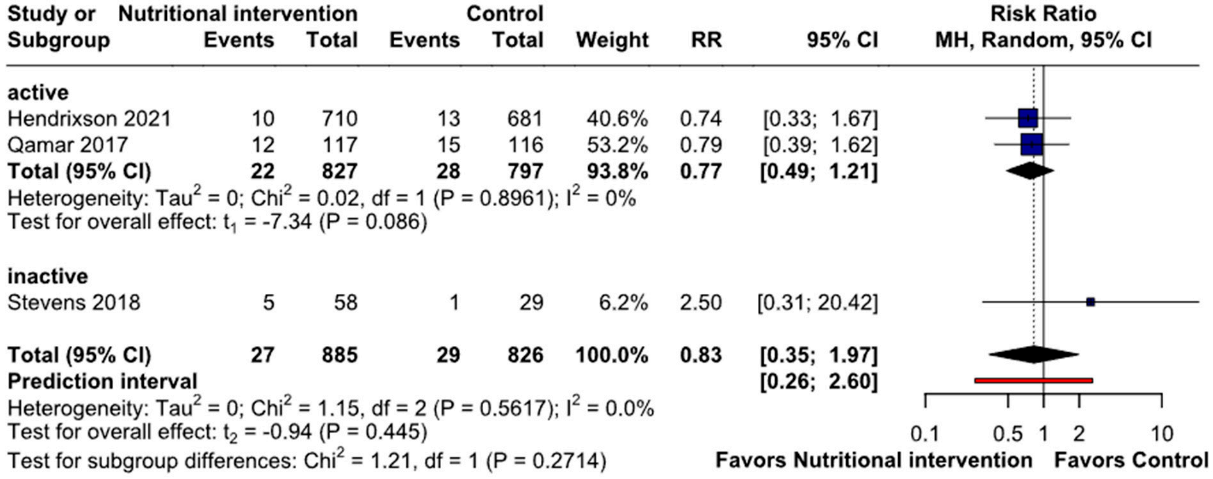

Figure S19. Subgroup forest plot of miscarriage according to comparator type, showing no significant effect in trials with active comparators or in the single trial with an inactive comparator, and no statistically significant between-subgroup difference.

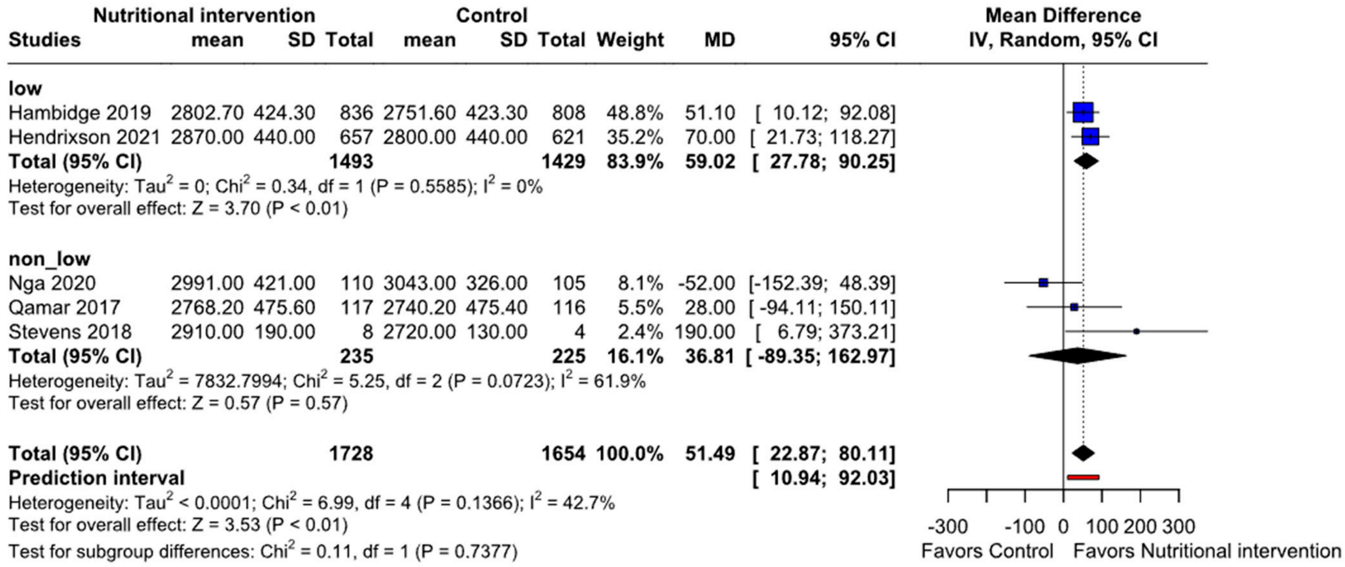

Figure S20. Sensitivity forest plot of birth weight stratified by overall risk of bias, showing a significant increase in low-risk studies but no significant effect in non-low-risk studies; the between-subgroup difference was not statistically significant.

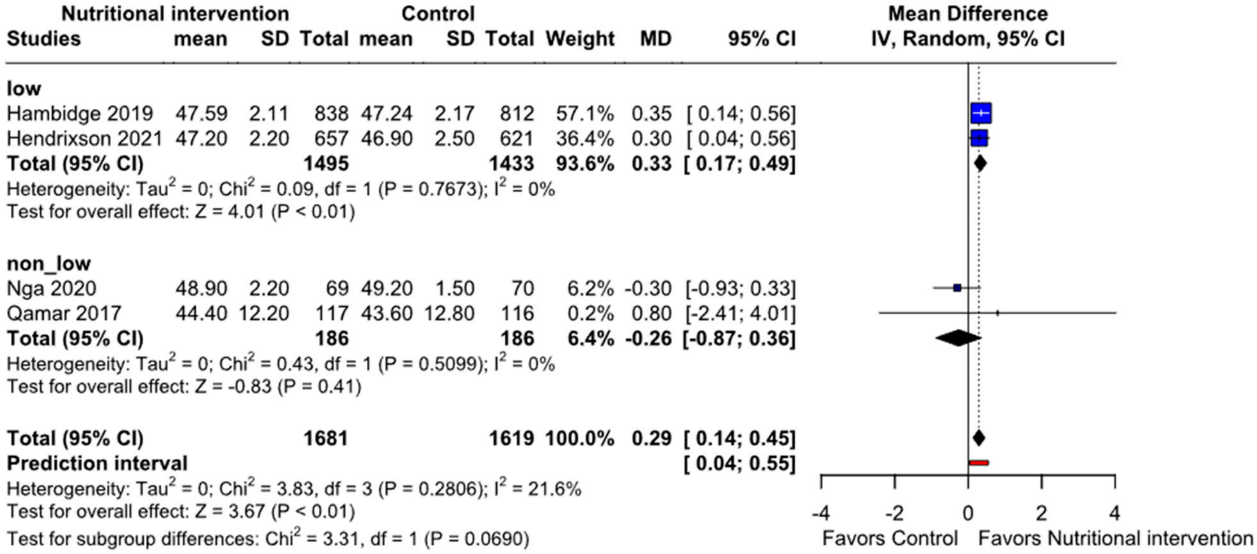

Figure S21. Sensitivity forest plot of birth length stratified by overall risk of bias, showing a significant increase in low-risk studies but no significant effect in non-low-risk studies; the between-subgroup difference did not reach statistical significance.

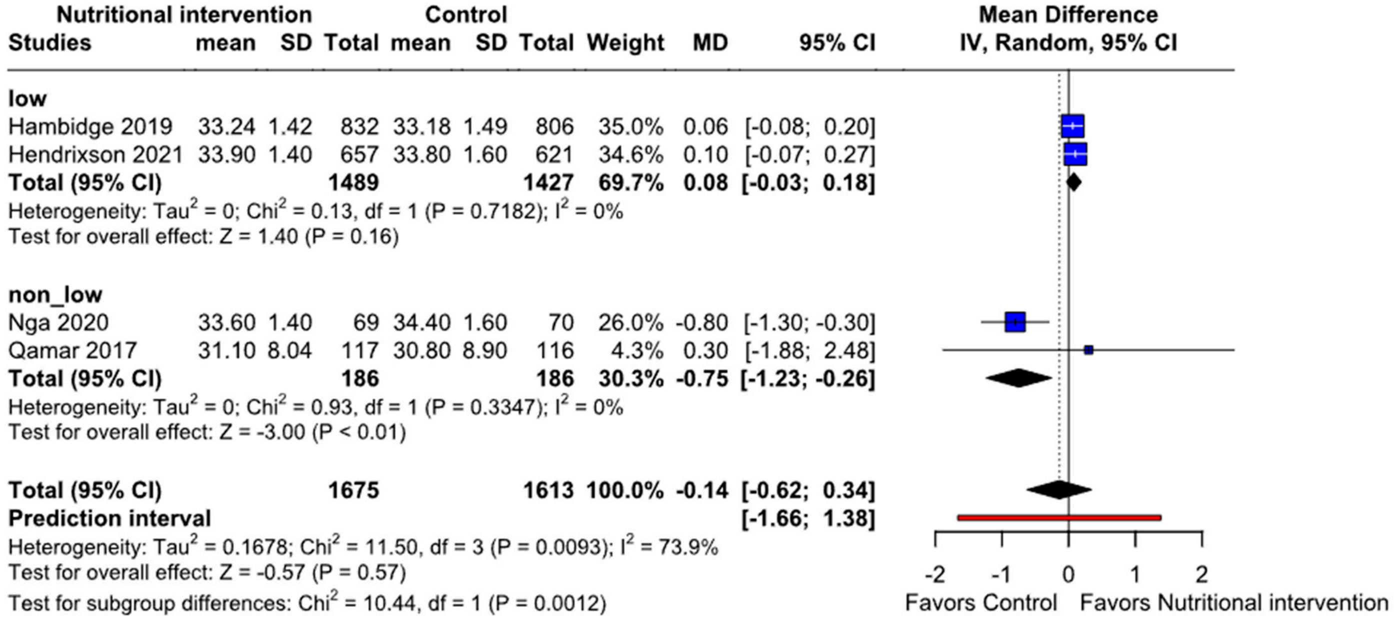

Figure S22. Sensitivity forest plot of birth head circumference stratified by overall risk of bias, showing no significant effect in low-risk studies but a significant reduction in non-low-risk studies; the between-subgroup difference was statistically significant.

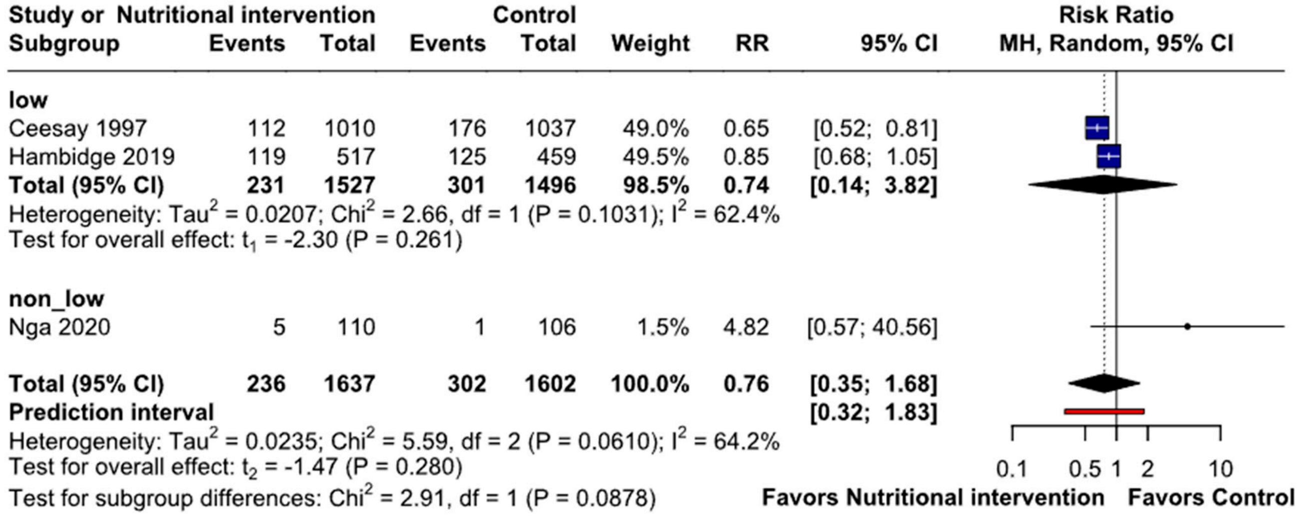

Figure S23. Sensitivity forest plot of low birth weight stratified by overall risk of bias, showing no significant effect in low-risk studies or in the single non-low-risk study, and no statistically significant between-subgroup difference.

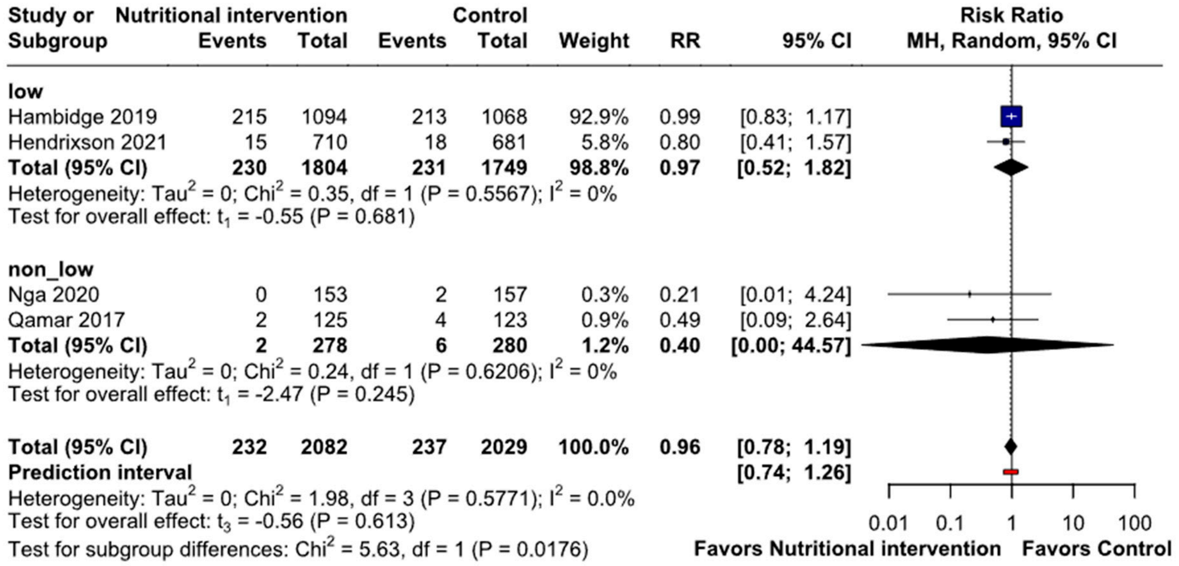

Figure S24. Sensitivity forest plot of stillbirth stratified by overall risk of bias, showing no significant effect within either risk group; although the between-subgroup difference was statistically significant, the non-low-risk estimate was extremely imprecise and should be interpreted cautiously.

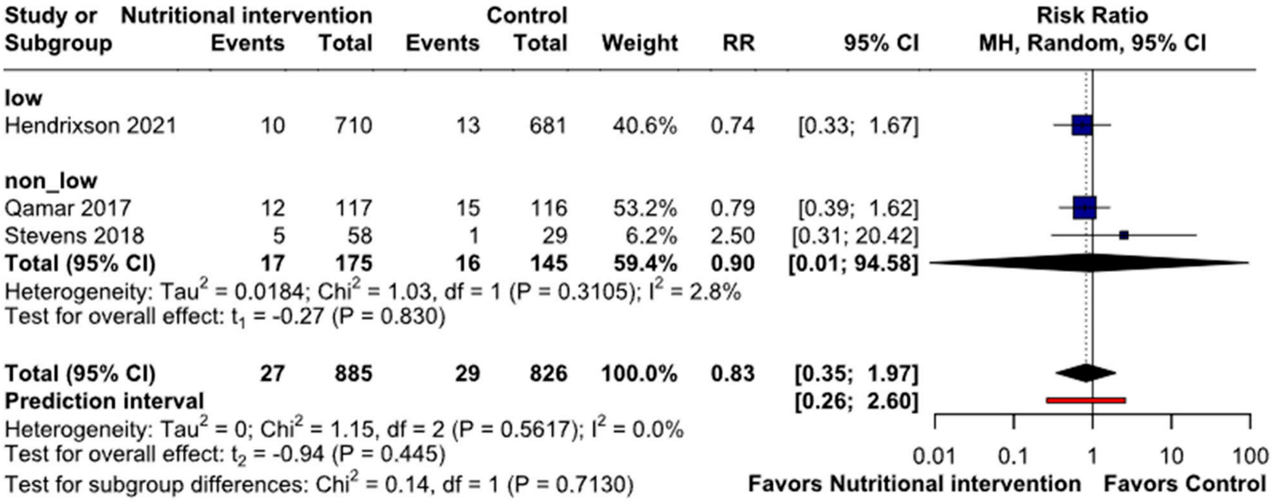

Figure S25. Sensitivity forest plot of miscarriage stratified by overall risk of bias, showing no significant effect in the single low-risk study or among non-low-risk studies, and no statistically significant between-subgroup difference.

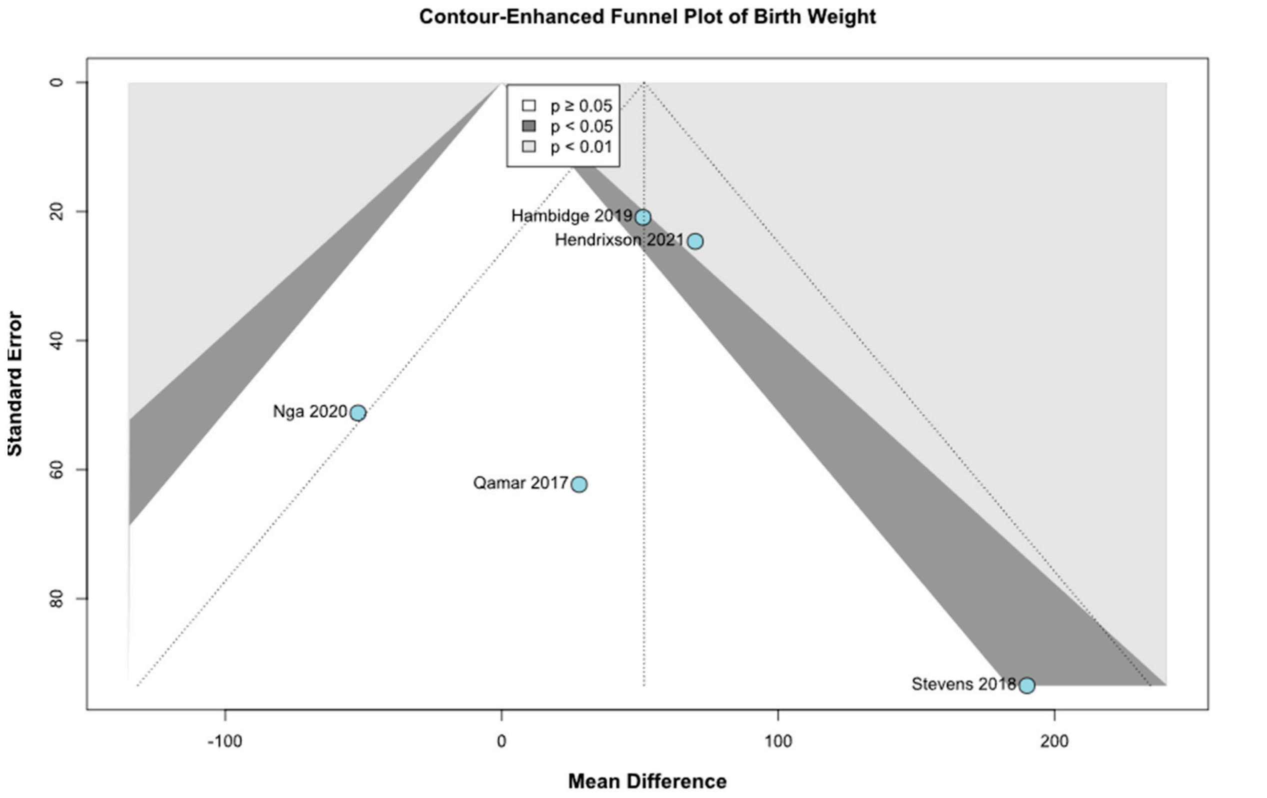

Figure S26. Contour-enhanced funnel plot for birth weight. Although some asymmetry is visually apparent, publication bias cannot be assessed reliably because fewer than 10 studies were included.

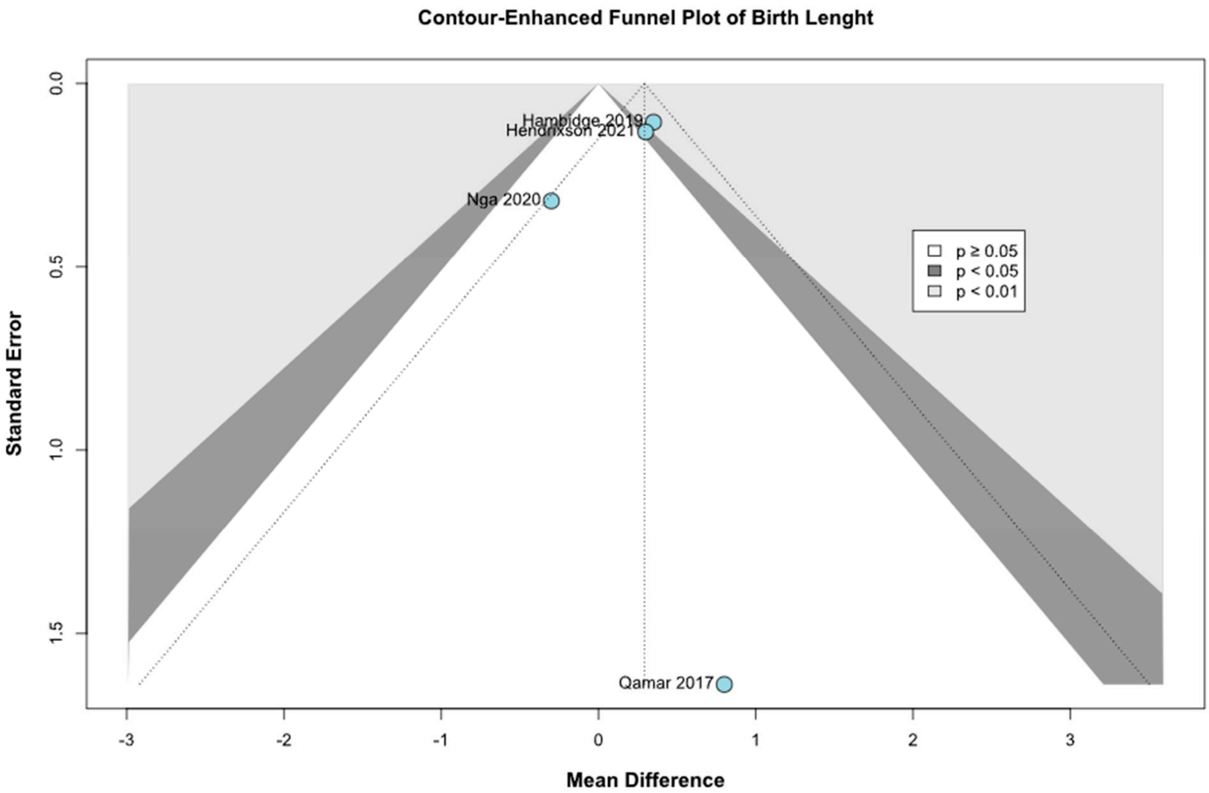

Figure S27. Contour-enhanced funnel plot for birth length. Visual asymmetry is apparent, but publication bias cannot be assessed reliably because only four studies were included.

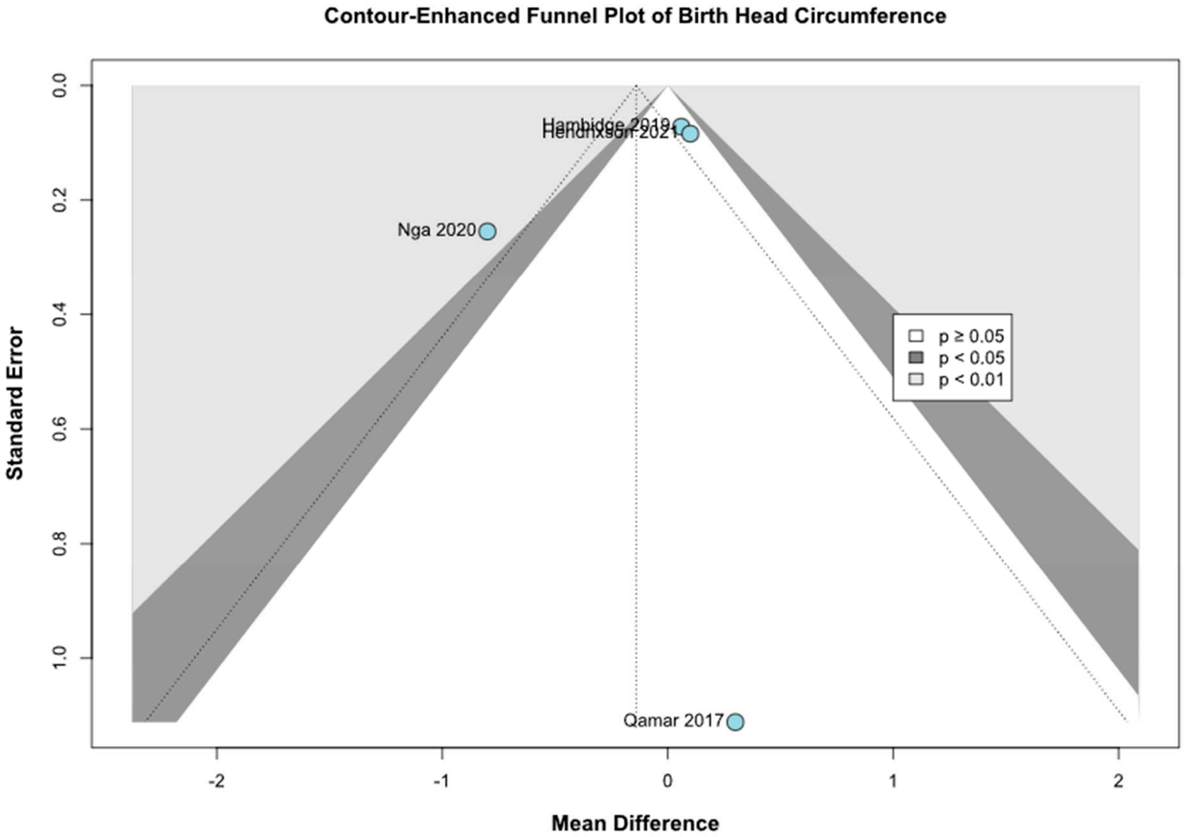

Figure S28. Contour-enhanced funnel plot for birth head circumference. Visual asymmetry is apparent, but publication bias cannot be assessed reliably because only four studies were included.

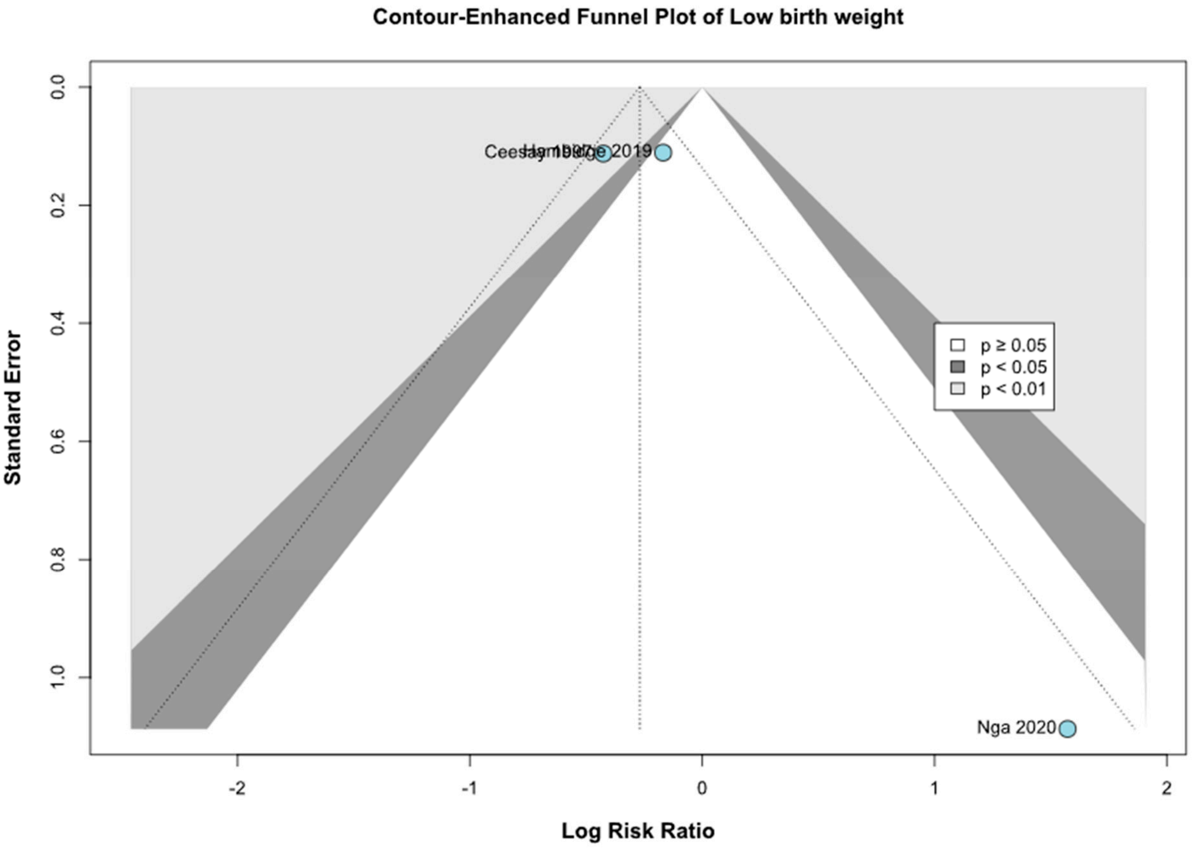

Figure S29. Contour-enhanced funnel plot for low birth weight. Visual asymmetry is apparent, but publication bias cannot be assessed reliably because only three studies were included.

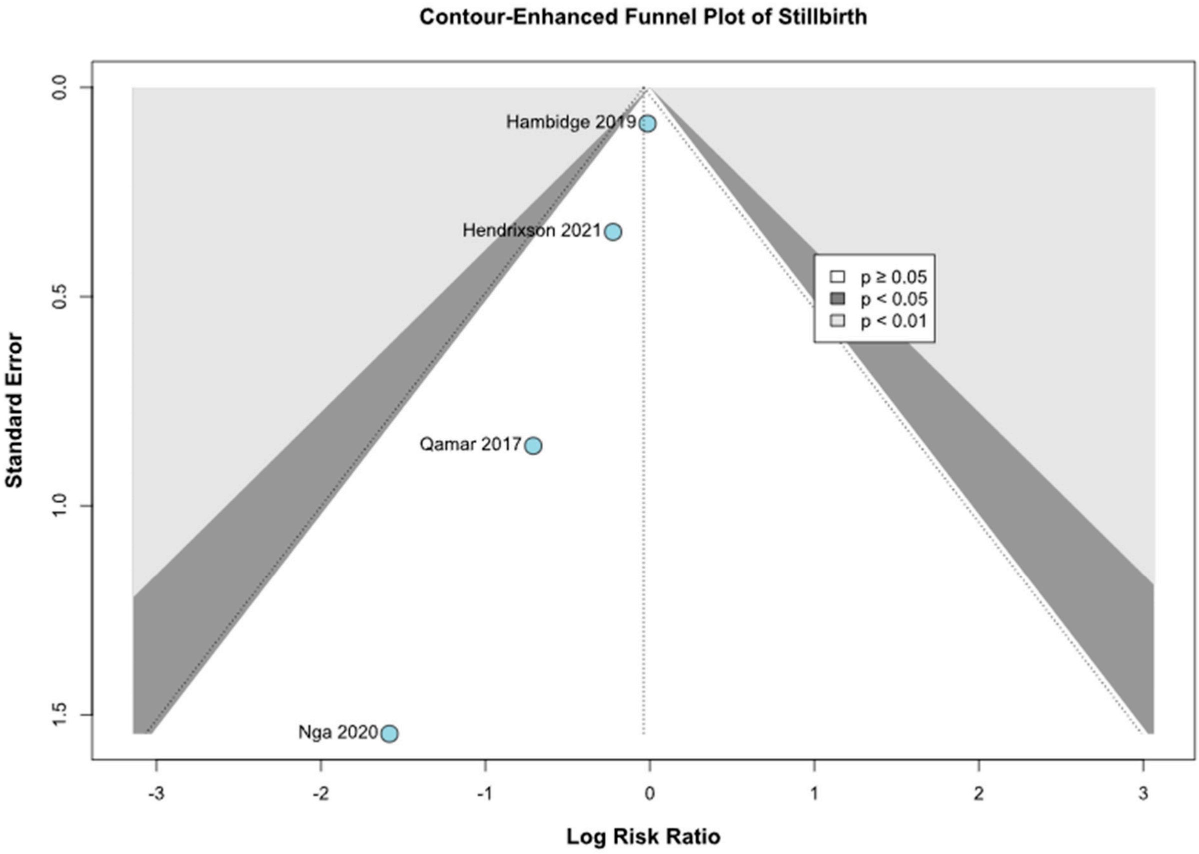

Figure S30. Contour-enhanced funnel plot for stillbirth. Some visual asymmetry is apparent, but publication bias cannot be assessed reliably because only four studies were included.

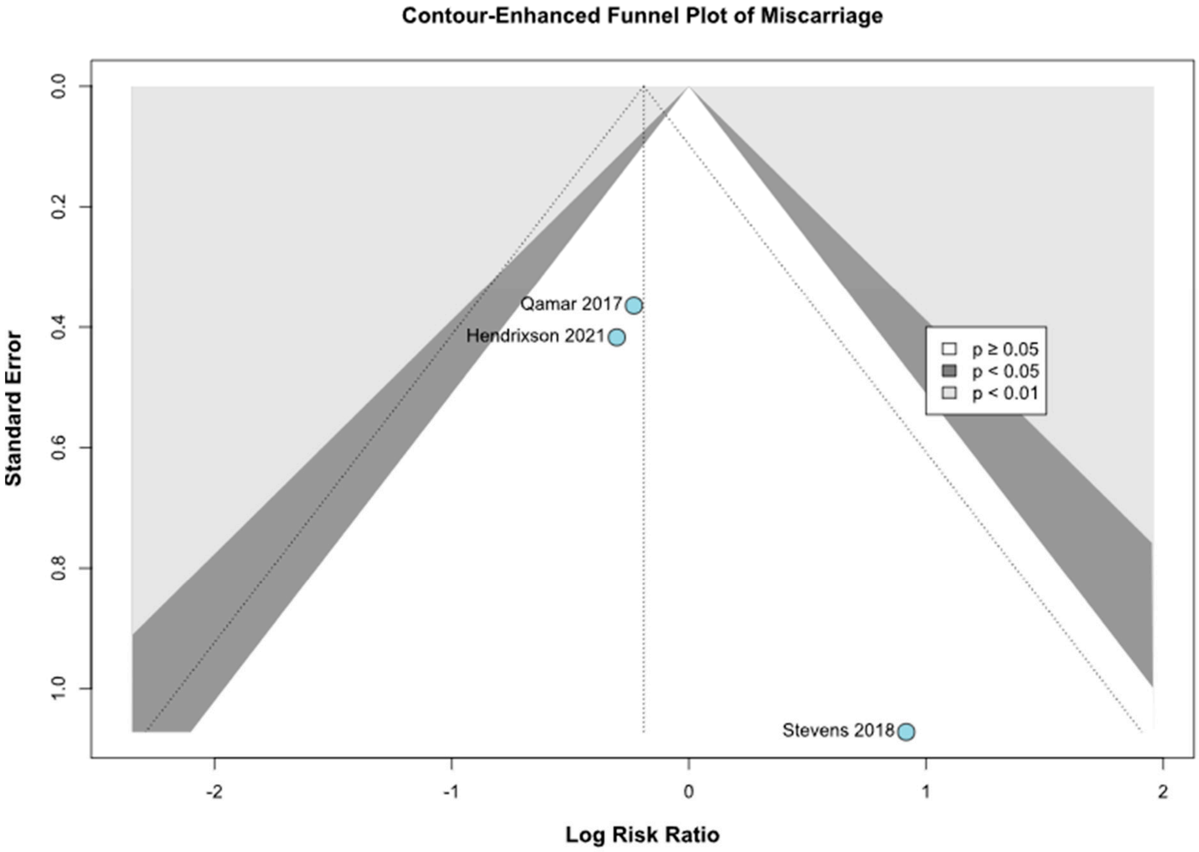

Figure S31. Contour-enhanced funnel plot for miscarriage. Visual asymmetry is apparent, but publication bias cannot be assessed reliably because only three studies were included.

PRISMA 2020 Checklist

Section and Topic	Item #	Checklist item	Location where item is reported
**TITLE**			
Title	1	Identify the report as a systematic review.	Title, 1-2
**ABSTRACT**			
Abstract	2	See the PRISMA 2020 for Abstracts checklist.	Abstract, 24-55
**INTRODUCTION**			
Rationale	3	Describe the rationale for the review in the context of existing knowledge.	Introduction, 56-80
Objectives	4	Provide an explicit statement of the objective(s) or question(s) the review addresses.	Introduction, 76-80
**METHODS**			
Eligibility criteria	5	Specify the inclusion and exclusion criteria for the review and how studies were grouped for the syntheses.	Methods, 87-97
Information sources	6	Specify all databases, registers, websites, organisations, reference lists and other sources searched or consulted to identify studies. Specify the date when each source was last searched or consulted.	Methods, 99-105
Search strategy	7	Present the full search strategies for all databases, registers and websites, including any filters and limits used.	Methods, 99-105
Selection process	8	Specify the methods used to decide whether a study met the inclusion criteria of the review, including how many reviewers screened each record and each report retrieved, whether they worked independently, and if applicable, details of automation tools used in the process.	Methods, 99-105
Data collection process	9	Specify the methods used to collect data from reports, including how many reviewers collected data from each report, whether they worked independently, any processes for obtaining or confirming data from study investigators, and if applicable, details of automation tools used in the process.	Methods, 99-105
Data items	10a	List and define all outcomes for which data were sought. Specify whether all results that were compatible with each outcome domain in each study were sought (e.g. for all measures, time points, analyses), and if not, the methods used to decide which results to collect.	Methods, 107-109
	10b	List and define all other variables for which data were sought (e.g. participant and intervention characteristics, funding sources). Describe any assumptions made about any missing or unclear information.	Methods, 107-109
Study risk of bias assessment	11	Specify the methods used to assess risk of bias in the included studies, including details of the tool(s) used, how many reviewers assessed each study and whether they worked independently, and if applicable, details of automation tools used in the process.	Methods, 111-118
Effect measures	12	Specify for each outcome the effect measure(s) (e.g. risk ratio, mean difference) used in the synthesis or presentation of results.	Methods, 119-150
Synthesis methods	13a	Describe the processes used to decide which studies were eligible for each synthesis (e.g. tabulating the study intervention characteristics and comparing against the planned groups for each synthesis (item #5)).	Methods, 119-150
	13b	Describe any methods required to prepare the data for presentation or synthesis, such as handling of missing summary statistics, or data conversions.	Methods, 119-150
	13c	Describe any methods used to tabulate or visually display results of individual studies and syntheses.	Methods, 119-150
	13d	Describe any methods used to synthesize results and provide a rationale for the choice(s). If meta-analysis was performed, describe the model(s), method(s) to identify the presence and extent of statistical heterogeneity, and software package(s) used.	Methods, 119-150

PRISMA 2020 Checklist

Section and Topic	Item #	Checklist item	Location where item is reported
	13e	Describe any methods used to explore possible causes of heterogeneity among study results (e.g. subgroup analysis, meta-regression).	Methods, 119-150
	13f	Describe any sensitivity analyses conducted to assess robustness of the synthesized results.	Methods, 119-150
Reporting bias assessment	14	Describe any methods used to assess risk of bias due to missing results in a synthesis (arising from reporting biases).	Methods, 111-118
Certainty assessment	15	Describe any methods used to assess certainty (or confidence) in the body of evidence for an outcome.	Methods, 111-118
**RESULTS**			
Study selection	16a	Describe the results of the search and selection process, from the number of records identified in the search to the number of studies included in the review, ideally using a flow diagram.	Results, 153-158
	16b	Cite studies that might appear to meet the inclusion criteria, but which were excluded, and explain why they were excluded.	Results, 153-158
Study characteristics	17	Cite each included study and present its characteristics.	Results, 159-160
Risk of bias in studies	18	Present assessments of risk of bias for each included study.	Results, 242-247
Results of individual studies	19	For all outcomes, present, for each study: (a) summary statistics for each group (where appropriate) and (b) an effect estimate and its precision (e.g. confidence/credible interval), ideally using structured tables or plots.	Results, 161-240
Results of syntheses	20a	For each synthesis, briefly summarise the characteristics and risk of bias among contributing studies.	Results, 161-240
	20b	Present results of all statistical syntheses conducted. If meta-analysis was done, present for each the summary estimate and its precision (e.g. confidence/credible interval) and measures of statistical heterogeneity. If comparing groups, describe the direction of the effect.	Results, 161-240
	20c	Present results of all investigations of possible causes of heterogeneity among study results.	Results, 161-240
	20d	Present results of all sensitivity analyses conducted to assess the robustness of the synthesized results.	Results, 161-240
Reporting biases	21	Present assessments of risk of bias due to missing results (arising from reporting biases) for each synthesis assessed.	Results, 242-247
Certainty of evidence	22	Present assessments of certainty (or confidence) in the body of evidence for each outcome assessed.	Results, 242-247
**DISCUSSION**			
Discussion	23a	Provide a general interpretation of the results in the context of other evidence.	Discussion, 249-300
	23b	Discuss any limitations of the evidence included in the review.	Discussion, 317-327
	23c	Discuss any limitations of the review processes used.	Discussion, 317-327

PRISMA 2020 Checklist

Section and Topic	Item #	Checklist item	Location where item is reported
	23d	Discuss implications of the results for practice, policy, and future research.	Discussion, 301-316
**OTHER INFORMATION**			
Registration and protocol	24a	Provide registration information for the review, including register name and registration number, or state that the review was not registered.	Methods, 83-86
	24b	Indicate where the review protocol can be accessed, or state that a protocol was not prepared.	Methods, 83-86
	24c	Describe and explain any amendments to information provided at registration or in the protocol.	Methods, 83-86
Support	25	Describe sources of financial or non-financial support for the review, and the role of the funders or sponsors in the review.	Under references, 568-569
Competing interests	26	Declare any competing interests of review authors.	Under references, 571
Availability of data, code and other materials	27	Report which of the following are publicly available and where they can be found: template data collection forms; data extracted from included studies; data used for all analyses; analytic code; any other materials used in the review.	Under references, 577

From: Page MJ, McKenzie JE, Bossuyt PM, Boutron I, Hoffmann TC, Mulrow CD, et al. The PRISMA 2020 statement: an updated guideline for reporting systematic reviews. BMJ 2021;372:n71. doi: 10.1136/bmj.n71
For more information, visit: http://www.prisma-statement.org/

Figure S32. PRISMA checklist
